# Modeling Local Aerosol Surface Environments: Clustering of Pyruvic Acid Analogs, Water, and Na^+^, Cl^–^ Ions

**DOI:** 10.1021/acsomega.4c09196

**Published:** 2025-01-02

**Authors:** Georg Baadsgaard Trolle, Jakub Kubečka, Jonas Elm

**Affiliations:** Department of Chemistry, Aarhus University, Langelandsgade 140, Aarhus C, Aarhus 8000, Denmark

## Abstract

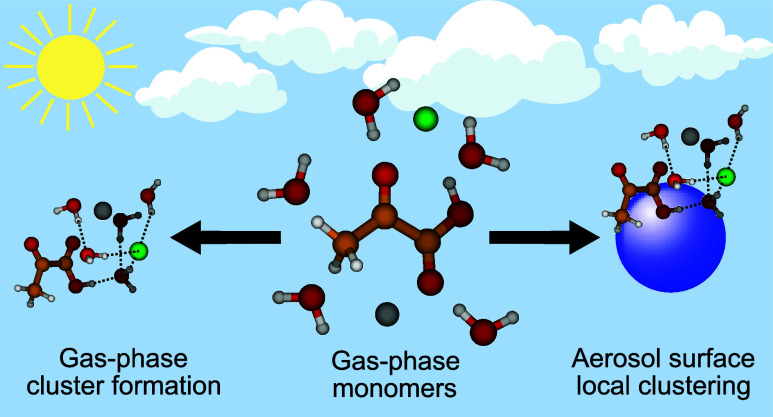

Pyruvic acid is an omnipresent compound in nature and is found both in the gas phase and in the particle phase of the atmosphere as well as in aqueous solution in the hydrosphere. Despite much literature on the photochemical degradation and stability of pyruvic acid in different chemical environments, the study of simultaneous interactions between gas-phase pyruvic acid or similar carboxylic acids with water and ions is not well-understood. Here, we present a study of microhydrated molecular clusters containing pyruvic acid and the structurally analogous carboxylic acids lactic acid, propionic acid, and 2,2-dihydroxypropanoic acid by probing geometries, binding free energies, hydrate distributions, as well as their infrared (IR) absorption spectra. We performed a meticulous configurational sampling protocol for the various hydrated clusters ranging from low level of theory to high level of theory to identify the lowest free energy structure. We find that cluster geometries and especially their water structure are highly sensitive to the presence and character of ions. We show that the hydration of the studied organic acids is thermodynamically unfavorable in the gas phase and ions are necessary for mediating interactions between organic acids and water thus stabilizing the clusters. Finally, we find a clear correlation between decreasing pyruvic acid carboxylic O–H stretching frequencies, increasing intensity when adding more water to the clusters, and a correlation between increasing redshifting of the O–H frequencies upon addition of ions to the clusters. The observations done in this study could pave the way to unravel the mechanisms behind the transitioning of organic acids from the gas phase to the particle phase.

## 1 Introduction

The small organic acid, pyruvic acid (PA), and its conjugate base, the pyruvate ion (PA^–^), are common chemical constituents of aerosols, fogs, clouds as well as seawater.^1,2^ PA is ubiquitous in the atmosphere and is emitted from both biogenic and anthropogenic sources and has been detected as a major component of secondary organic aerosols (SOA) at many geographic locations.^3,4^ These include the urban atmospheres of Japan,^5^ China,^6−8^ and Mongolia^9^ but PA has also been detected in remote aerosols at mountaintops in China,^3^ tropic Indian aerosols,^10^ marine aerosols,^11−13^ arctic aerosols,^14^ and even in biomass burning aerosols in Brazil.^15^ The particle-phase concentration of PA has been observed to possess a large spatiotemporal variability with average particle-phase concentrations measured as high as 49.7 ng m^–3^ in secondary organic aerosols (SOA) of the urban atmosphere of Tokyo, Japan and as low as 0.03 ng m^–3^ in marine aerosols over the Southern Ocean.^5,12^ Furthermore, particle-phase concentrations of PA are observed to fluctuate significantly depending on season.^4,5^

In the aqueous phase, PA has been subjected to many photochemical investigations and has been reported to produce acetoin, lactic acid, and acetic acid and possibly also contribute to SOA mass through an oligomerization process, which goes via the parapyruvic acid dimer.^16−20^ Furthermore, PA has been found to exist as a mixture of the keto form and its hydrate, the geminal diol 2,2-dihydroxypropanoic acid (termed diol in this study).^20,21^ Hence, PA interconverts between the diol, the conjugate base of the diol, and the conjugate base of PA (PA^–^) in a cyclic manner.^20^ The ratio of abundances of the keto form and the diol has previously been reported to be 65% diol and 35% ketone in aqueous solution, at 298 K and neutral pH.^16,20,22^ Furthermore, the p*K*_a_ of PA in its keto form in aqueous solution and at 298 K is determined to be 2.18 while for the diol, it is determined to be 3.6.^20^ In perspective, aerosols are usually acidic with a median pH of 2.5, which implies that PA in its keto form is the most favored in aerosols at normal ambient conditions.^23^ In addition to the pure aqueous-phase studies of PA, there has also been conducted several photochemical experiments of PA in e.g., multiphase environments and at air–water interfaces in order to probe the dynamic equilibrium between PA in the aqueous phase and PA in the gas phase.^24,25^

PA has also been studied extensively in the gas phase.^2,26−30^ The gas-phase mixing ratio of PA has mainly been measured in the range 10–100 pptv but has been detected as high as 400 pptv in the Amazonas region of Brazil and in the southern US.^2,27,30^ The atmospheric sources of PA in the troposphere are manifold and the routes to its formation go mainly through gas-phase photolysis of small organic precursors. This includes the photo-oxidation of isoprene via the ozonolysis of methyl vinyl ketone,^31−33^ the photolysis of methylglyoxal,^34^ the reactions of peroxy radicals formed in the oxidation of propane, acetone, and hydroxyacetone,^35,36^ and in the photo-oxidation of aromatic compounds in the presence of NO_*x*_.^37,38^ The atmospheric sinks of PA, on the other hand, are generally 4-fold: (i) By rapid gas-phase photolysis induced by actinic radiation;^39^ (ii) by slow gas-phase reaction with OH radicals;^2^ (iii) by wet and dry deposition, and (iv) by partitioning into the aerosol particle phase.^16−18,40−43^ The atmospheric fate of PA via photolysis proceeds primarily via an exothermic decarboxylation, which involves a five-membered transition state that decomposes into CO_2_ and methylhydroxycarbene with the latter rapidly rearranging to acetaldehyde.^2,30^ The gas-phase lifetime of PA with respect to the photolysis channel is a few hours according to previous experimental studies.^44,45^ Conversely, the gas-phase lifetime of PA against the OH channel is significantly longer at about three months.^46^ The atmospheric fate of PA via the dry and wet deposition mechanisms as well as by the partitioning into the aerosol phase are particularly pronounced at high relative humidities due to the high solubility of PA in water and can thus contribute to the formation of SOA.^16−18,40−43^

While the gas-phase and aqueous-phase chemistry of PA has been extensively studied, the transition between the two has not yet been investigated in detail. The uptake of PA onto aerosol particles must occur via the interfacial surface layer. As a first approximation the local environment of the interaction between the gas-phase PA and the surface can be viewed as a small cluster of molecules consisting of water and ions. Despite previous studies on the PA monohydrate cluster, the diol in the liquid phase^21^ and the PA clusters with one to four water molecules,^47^ there has, to the best of our knowledge, not been reported any studies on how PA or similar carboxylic acids simultaneously interact with water molecules and ions.

In this study, we investigate clusters of PA and its structurally similar analogs, lactic acid (LA), propionic acid (ProA), and 2,2-dihydroxypropionic acid (diol), by probing the geometries and computing cluster binding free energies, hydrate distributions, and infrared (IR) absorption spectra. We specifically look at the intermolecular interactions between the organic acids, water (w) and ions (Na^+^, Cl^–^ and a NaCl pair). By viewing such clusters as a representation of the local aerosol surface environment, our aim is to elucidate how such small organic acids interact with the surface and ultimately how they enter the particle.

## 2 Computational Details

Initial configurational sampling was performed with the ABCluster program version 2.0, using a CHARMM force field.^48,49^ Subsequent single-point energies, geometries, and configurational sampling were calculated employing the GFN1-xTB method^50^ in the xtb software versions 6.4.0 and 6.4.1.^51^ Gaussian 16 version B.01 was utilized for the density functional theory (DFT) geometry optimizations and vibrational frequency calculations at the ωB97X-D/6-31++G(d,p) level of theory.^52^ ORCA version 5.0.4 was employed for calculating high level single-point energies using the DLPNO–CCSD(T_0_)/aug-cc-pVTZ methodology.^53^ The DLPNO–CCSD(T_0_)/aug-cc-pVTZ//ωB97X-D/6-31++G(d,p) level of theory has been thoroughly benchmarked in the literature and found the most optimal for studying the binding energetics of atmospheric molecular clusters.^54−57^ The choice is corroborated by additional benchmarking of the PA structures and interaction energies carried out in this work (see Supporting Information). The above software was used as third-party programs implemented in the JKCS program, which fully automate the cluster configurational sampling.^58^ The coordinates of the calculated structures and the associated thermochemistry are available in the Atmospheric Cluster Database^59^ (see Supporting Information).

### 2.1 Cluster Systems

**Figure 1:**
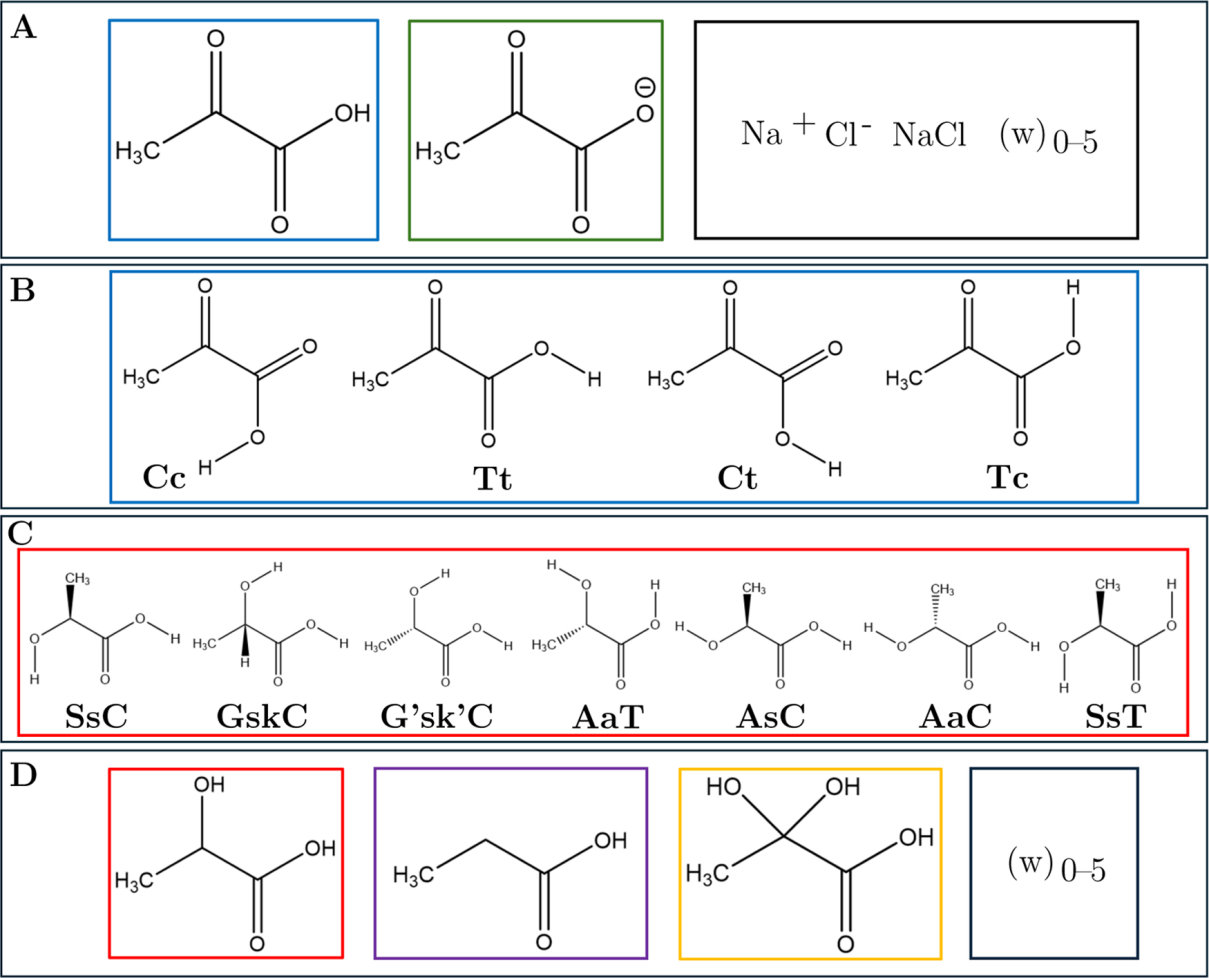
Overview of the various organic acids probed in this study. (A) PA (blue frame), PA^–^ (green frame), and ions with water (w) (black frame); (B) the four conformers of PA termed according to the cis–trans nomenclature;^60^ (C) the seven conformers of LA assigned conformational abbreviations in conformity with the nomenclature of Borba et al.;^61^ (D) LA (red frame), ProA (purple frame), diol (yellow frame), and water (black frame). Note that there are two conformers of ProA corresponding to an eclipsed and a staggered conformer, which are not represented here. Note also that there are four conformers of the diol but as these are equivalent to the conformers of PA, they are omitted for clarity.

We studied the (PA)_1_(w)_0–5_, (LA)_1_(w)_0–5_, (ProA)_1_(w)_0–5_, (diol)_1_(w)_0–5_, (PA)_1_(Na^+^)_0–1_(Cl^–^)_0–1_(w)_0–5_, (PA^–^)_1_(w)_0–5_ and the (PA^–^)_1_(Na^+^)_1_(Cl^–^)_0–1_(w)_0–5_ cluster systems. Figure 1B presents the four different conformers of PA.^60^ The PA conformers are conventionally assigned conformational abbreviations according to the cis–trans nomenclature, with the first letter denoting the geometric relationship between the two carbonyl groups and the second letter denoting the geometric relationship between the α-carbonyl group and the carboxylic acid proton.^60^ Blair et al. calculated the relative zero-point vibrationally corrected electronic energies of the four PA conformers to be 0.00 kcal/mol for the **Tc** conformer, 2.68 kcal/mol for the **Tt** conformer, 4.36 kcal/mol for the **Ct** conformer, and 10.31 kcal/mol for the **Cc** conformer at 298 K and at the ωB97X-D/aug-cc-pVTZ level of theory.^60^

In addition to the conformers of PA, Figure 1A and Figure 1D provide a general overview of the clusters and monomers probed in this study and Figure 1C provides the conformers of LA.

### 2.2 Configurational Sampling

We employed a well-established, extensive configurational sampling (CS) protocol to obtain the cluster structures. The ABCluster input files for PA, LA, ProA, and the diol were generated with topgen.^62^ Hirshfeld population analysis was calculated at the MP2/6-31++G(d,p) level of theory.^63^ We used the Charge Model 5 (CM5), which has a reduced basis set sensitivity and geometry dependency.^64^ The overall configurational sampling workflow can be described as follows
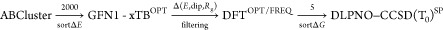
1

The ABCluster search was performed based on the parameters recommended by Kubečka et al., with a population of SN = 300 times the number of molecules in the cluster, number of generations *g*_max_ = 200, and a maximal structure lifetime of sc = 4 generations for all cluster sizes.^65^ All protonation states of water (H_2_O, H_3_O^+^, and OH^–^) leading to electrical neutral clusters and all conformers of PA, LA, ProA, and the diol were employed in the ABCluster search. The overall number of local minima saved for subsequent GFN1-xTB calculations was 2000 (lm = 2000/NS, where NS refers to the total number of simulations) and were sorted with respect to electronic energy.

The selected structures were preoptimized at the GFN1-xTB level.^50^ After the preoptimization, a filtering maneuver was performed with respect to the collective variables: Electronic energy (0.001 Ha cutoff), radius of gyration (0.01 Å cut-off), and electric dipole moment (0.1 D cutoff) to identify unique configurations.

After filtering, full geometry optimization and vibrational frequency calculations were performed at ωB97X-D/6-31++G(d,p) level of theory for all the unique GFN1-xTB structures.^52^ Structures where the lowest vibrational frequencies were negative and structures, which did not converge were resubmitted for further optimization. After all DFT calculations were finished, the quasi-harmonic approximation was applied using a frequency cutoff at 100 cm^–1^.^66,67^ All frequencies below this cutoff are treated as free rotations when calculating the entropy. We note that we neglect anharmonicity in our calculations. A common practice is to apply vibrational frequency scaling factors. However, to the best of our knowledge, except for pure water clusters,^68−70^ there exist no accurate scaling factors derived for general use for atmospherically relevant clusters. Finally, single-point electronic energies were calculated with the DLPNO–CCSD(T_0_) method on the five lowest Gibbs free energy structures.^71,72^ For these calculations, the RI-JK approximation with the aug-cc-pVTZ basis set, and with an auxiliary basis set for the Coulomb and exchange parts was applied together with the TightSCF convergence criterion.

#### 2.2.1 Calculations of Binding Free Energies

The binding free energy (Δ*G*_bind_) of the clusters is defined as
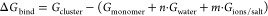
2

where *G*_monomer_ corresponds to the Gibbs free energy of the organic acid monomer, *G*_water_ corresponds to the Gibbs free energy of a water molecule, *G*_ions/salt_ corresponds to the Gibbs free energy of the Na^+^ or Cl^–^ ion or the NaCl pair, and *n* and *m* correspond to the number of water molecules and the number of ions in the cluster, respectively. We calculate the Gibbs free energy for each cluster component as a combination of a high-level electronic contribution and a DFT thermal free correction:
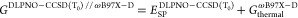
3Here the thermal free energies are calculated at 298.15 K and 1 atm. Thermal free energies at other temperatures are calculated based on the assumption that the enthalpy and entropy does not change significantly when changing the temperature. The calculated binding free energies employing eq 3 should lead to errors on the order of 1–2 kcal/mol or below.^73^

#### 2.2.2 Calculations of Hydrate Distributions

For the hydrate distributions presented in this study, we apply the following equation obtained from statistical mechanics and classical nucleation theory
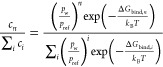
4where *c*_*n*_ is the concentration (or probability) of a cluster of size *n*, *c*_*i*_ is the concentration (or probability) of a cluster component *i*, *p*_w_ and *p*_ref_ are the partial pressure of water and the equilibrium saturation vapor pressure of water, respectively, Δ*G*_bind,*n*_ is the binding free energy associated with forming a cluster of size *n*, Δ*G*_bind,*i*_ is the binding free energy associated with the cluster component, *i*, *k*_B_ is the Boltzmann constant, and *T* is the temperature. The hydrate distribution shows the population (in percentage) of each cluster system. It essentially shows the fractional number of water molecules that are bound to the cluster, i.e., the probability that a given number of water molecules are bound.

### 2.3 IR Absorptions of Pyruvic Acid Clusters

Multiple experimental studies have been reported on the H-shift for the intramolecular hydrogen bond between the carboxylic O–H bond and the α-carbonyl group of PA.^28,29^ Thus, vibrational spectroscopy has been an important tool in distinguishing between the various conformers of PA.

In order to computationally probe the hydrogen bond shift of the PA carboxylic O–H bond in the clusters, we calculated the harmonic IR spectra of the various PA-containing clusters. GaussView version 6.0.16^74^ was employed for visually inspecting the vibrational normal modes of the clusters and to identify the relevant carboxylic O–H stretching mode. The discrete vibrational modes for the global minimum structure of each cluster corresponding to the carboxylic O–H stretching mode were then collected and presented as an IR spectrum.

## 3 Results and Discussion

### 3.1 Cluster Geometries

All cluster geometries presented in this section were obtained at the DLPNO–CCSD(T_0_)/aug-cc-pVTZ//ωB97X-D/6-31++G(d,p) level of theory, at 298.15 K and 1 atm using the quasi-harmonic approximation.

#### 3.1.1 Reference Systems

**Figure 2:**
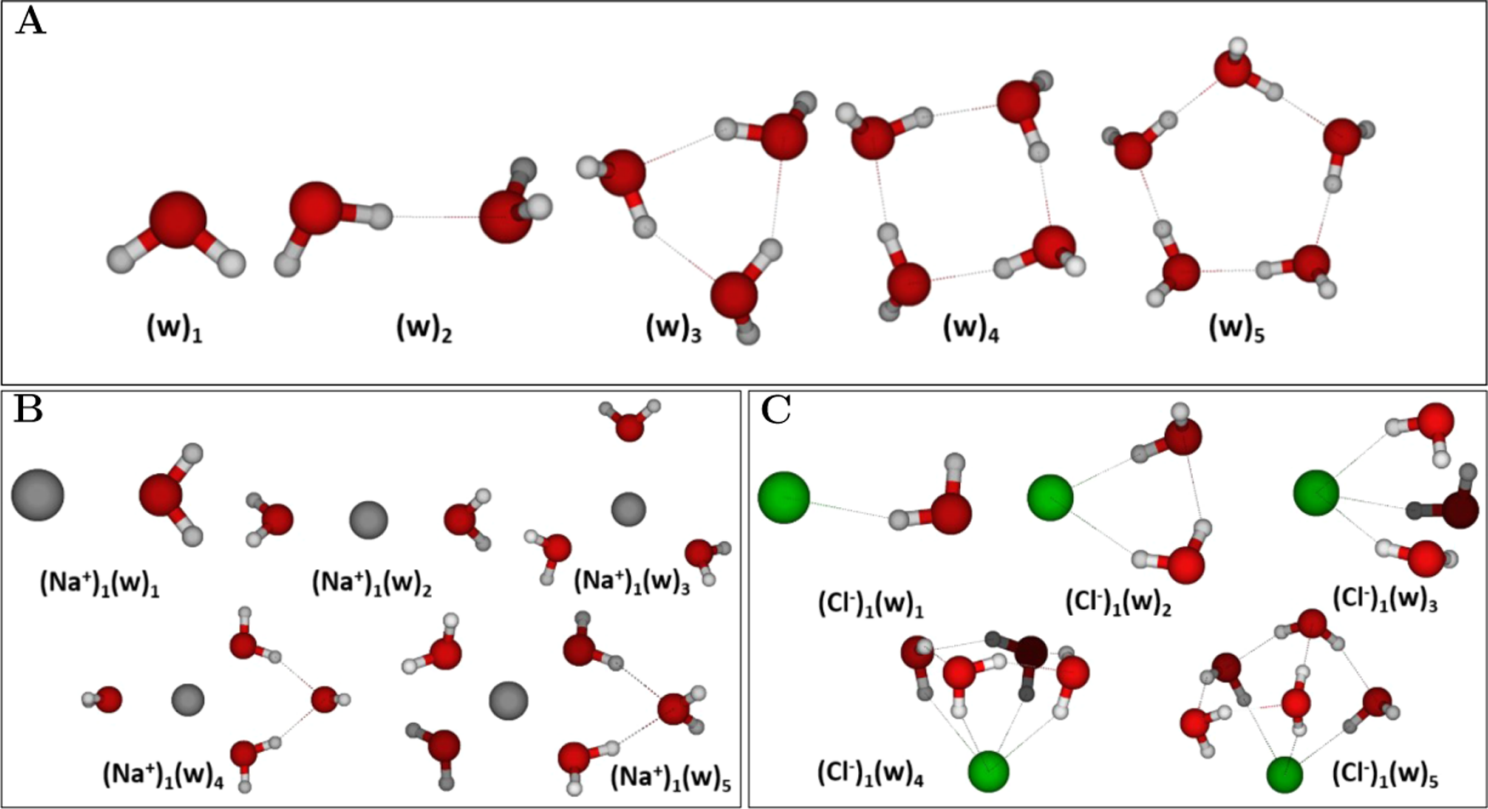
Lowest free energy clusters of water (w) and microhydrated Na^+^ and Cl^–^ions: (A) (w)_1–5_; (B) (Na^+^)_1_(w)_1–5_; (C) (Cl^–^)_1_(w)_1–5_. The geometries were obtained at the DLPNO–CCSD(T_0_)/aug-cc-pVTZ//ωB97X-D/6-31++G(d,p) level of theory, at 298.15 K and 1 atm using the quasi-harmonic approximation. Hydrogen atoms are white, oxygen red, sodium gray, and chlorine green.

To test our configurational sampling methodology, we initially studied the simple (w)_1–5_, (Na^+^)_1_(w)_1–5_, and (Cl^–^)_0–1_(w)_1–5_ cluster systems. We obtained the cluster geometries shown in Figure 2.

Multiple experimental studies have reported the gas-phase microhydration of the sodium ion^75−81^ along with computational investigations.^82−102^ In a similar manner, the microhydration of the chloride ion has also been studied experimentally,^81,103,104^ in addition to computational investigations.^84,85,88,89,98,104−106^ Generally, we see high similarity between our geometries at the given level of theory and literature geometries obtained at the MP2/aug-cc-pVDZ level of theory for the hydrated gas-phase clusters of a sodium ion^102^ and a chloride ion.^98^ In a similar manner, our identified (w)_1–5_ cluster geometries seen in Figure 2A follow the conventional structure of pure gas-phase water clusters reported in the literature.^107−109^

Figure 2B shows the (Na^+^)_1_(w)_1–5_ cluster geometries. For (Na^+^)_1_(w)_1_, we observe an ion-dipole interaction. For (Na^+^)_1_(w)_2_, we see a linear assembly of water molecules with the Na^+^ ionic center. For (Na^+^)_1_(w)_3_, the cluster adopts a trigonal planar geometry of the waters surrounding the Na^+^ center. For the (Na^+^)_1_(w)_4_ cluster, a quasi-square planar geometry is seen due to the additional water molecule (confirmed with literature geometries^89,92,102^) but is also reported to be able to adopt a tetrahedral geometry.^89,92,102^ For (Na^+^)_1_(w)_5_, four water molecules are interacting with Na^+^ and one water resides at the periphery of the cluster, which corresponds to the most reported literature geometries even though square pyramidal geometries also occur.^89,92,102^

Figure 2C presents the (Cl^–^)_1_(w)_1–5_ cluster geometries. For (Cl^–^)_1_(w)_1_, we observe a polarization-enhanced hydrogen bond. For (Cl^–^)_1_(w)_2_, we see a trigonal planar geometry of all components without an ionic center (in contrast to the (Na^+^)_1_(w)_3_ cluster described above). For (Cl^–^)_1_(w)_3_, the cluster adopts a trigonal pyramidal geometry around the central Cl^–^. For the (Cl^–^)_1_(w)_4_ cluster, a square pyramidal geometry around the central Cl^–^ is seen. For (Cl^–^)_1_(w)_5_, a quasi-square pyramidal geometry around the central Cl^–^ is observed, which possess a slight deviation from the four-water planarity observed in the (Cl^–^)_1_(w)_4_ cluster. In general, the (Cl^–^)_1_(w)_1–5_ clusters are also in full agreement with literature geometries.^98,104,106^

Overall, Na^+^ is observed to be encapsulated inside the clusters and solvated by the surrounding water molecules. In contrast, Cl^–^ is residing at the edge of the clusters. We argue that the electrostatic repulsions experienced by Cl^–^ due to the presence of electronegative oxygen atoms in water and the spatial uptake necessary for Cl^–^ to be encapsulated in the clusters altogether entail that it will rather reside at the edge of the clusters.

#### 3.1.2 Hydration of Organic Acids

**Figure 3:**
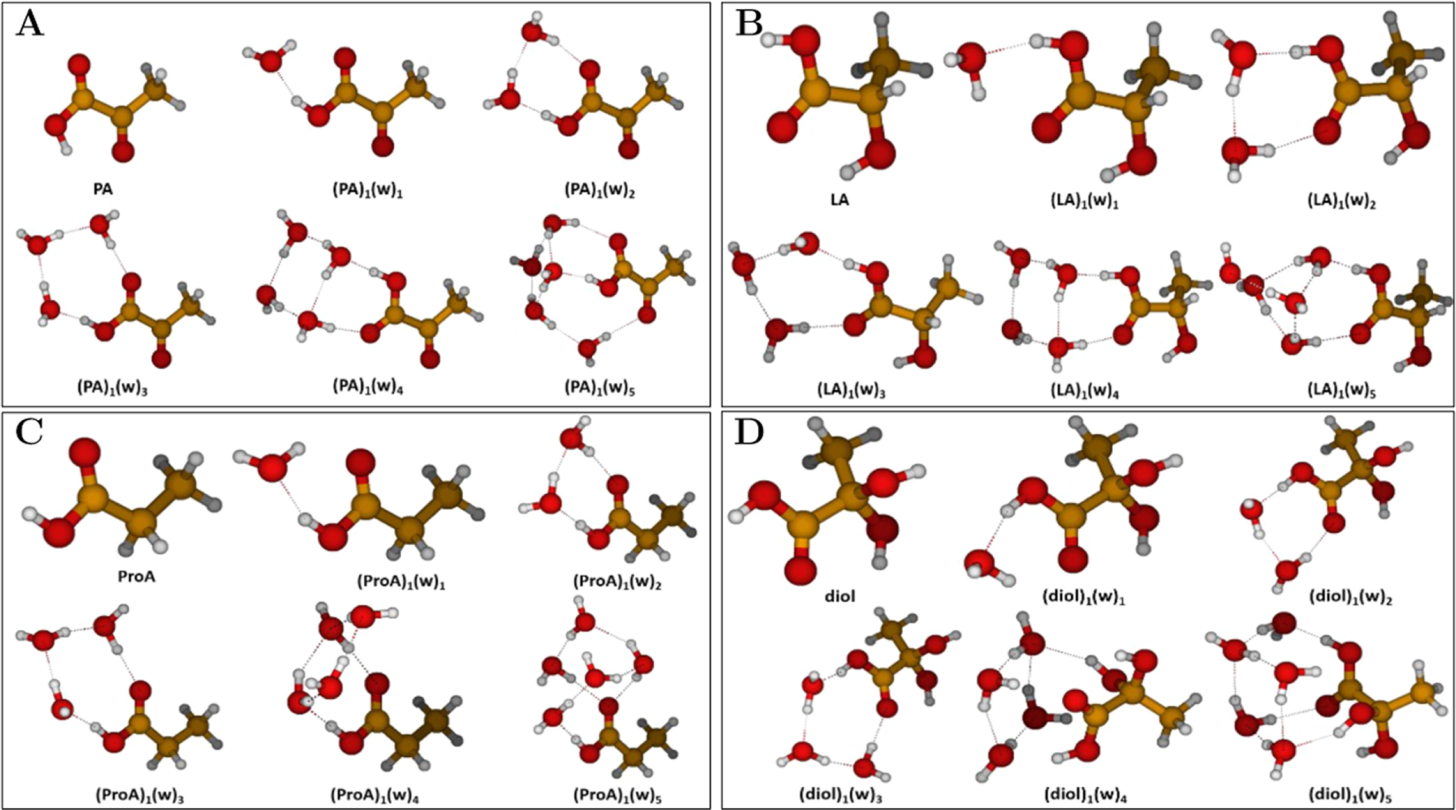
Lowest free energy clusters of PA, LA, ProA, and diol with up to five water (w) molecules: (A) (PA)_1_(w)_0–5_; (B) (LA)_1_(w)_0–5_; (C) (ProA)_1_(w)_0–5_; (D) (diol)_1_(w)_0–5_. The geometries were obtained at the DLPNO–CCSD(T_0_)/aug-cc-pVTZ//ωB97X-D/6-31++G(d,p) level of theory, at 298.15 K and 1 atm using the quasi-harmonic approximation. Hydrogen atoms are white, carbon brown, and oxygen red.

Using the outlined configurational sampling protocol we studied the (PA)_1_(w)_0–5_, (LA)_1_(w)_0–5_, (ProA)_1_(w)_0–5_, and (diol)_1_(w)_0–5_ cluster systems. Figure 3 presents the hydrated cluster geometries of PA (Figure 3A), LA (Figure 3B), ProA (Figure 3C), and the diol (Figure 3D).

In no cases do we observe a proton transfer from the organic acids to water. We argue that the low acidity of the organic acids studied here implies that significantly more surrounding water molecules are required in the clusters in order to observe proton transfer events. As seen in Figure 3A, the **Tc** conformer of PA was identified as the lowest free energy conformer, as expected. However, when hydrating the monomer, it takes the form of the **Tt** conformer within the (PA)_1_(w)_1–3_ and (PA)_1_(w)_5_ clusters. This can be ascribed to the PA monomer engaging in favorable hydrogen-bonding interactions with the surrounding water molecules thus impeding intramolecular hydrogen bonding between the carboxylic O–H donor and the α-carbonyl acceptor. In the (PA)_1_(w)_4_ cluster, the **Ct** conformer of PA is observed instead but the configuration of the carboxylic acid moiety is retained as the **Ct** conformer is simply generated by a 180-degree rotation around the C–C bond between the two carbonyl groups of the **Tc** conformer. Generally, the water molecules are observed to reside at the carboxylic acid terminus of the PA monomer and the hydrogen bonding capacity of the carboxylic oxygens are observed to be saturated at the (PA)_1_(w)_4_ cluster, i.e., the fifth water molecule in the (PA)_1_(w)_5_ cluster engages in hydrogen bonding with the α-carbonyl group instead, thus serving as a water bridge to the adjacent four-water ensemble.

When comparing the PA clusters with literature, we find that the (PA)_1_(w)_1_ cluster is consistent with the cluster obtained by Petersen-Sonn et al.^47^ at CAM-B3LYP/aug-cc-pVTZ level of theory and by Shemesh et al.^110^ at MP2/cc-pVDZ level of theory. However, for the (PA)_1_(w)_2–5_ clusters we find slight discrepancies between our geometries and the literature geometries as the water structure here is much more diffuse compared to our well-centered and well-organized water structure. These geometric discrepancies could be ascribed to both differences in configurational sampling protocol and level of theory.

In case of the PA analogs in Figure 3B–D, we observe very similar geometrical patterns as for the PA clusters in Figure 3A. For the (LA)_1_(w)_0–5_ clusters, the SsC conformer of LA^61^ is the lowest free energy conformer across all clusters due to the intramolecular hydrogen bond between the α-hydroxy group and the carboxylic carbonyl group as well as the minimization of steric repulsions induced by the methyl group as it protrudes away from the carboxylic acid plane of LA. For the (ProA)_1_(w)_0–5_ clusters, ProA takes the planar and entirely staggered conformer over the bent conformer across all clusters. For the (diol)_1_(w)_0–5_ clusters, the **Ct** conformer dominates across all clusters due to the intramolecular hydrogen bond between the carboxylic carbonyl group and one of the α-hydroxy groups while the other α-hydroxy group and the methyl group protrude away from the main carboxylic acid plane of the diol.

In general, for all organic acid clusters containing two and four water molecules in Figure 3, we observe a conservation of the water structure with respect to the pure water clusters presented in Figure 2A.

#### 3.1.3 Pyruvic Acid-Containing Clusters

**Figure 4:**
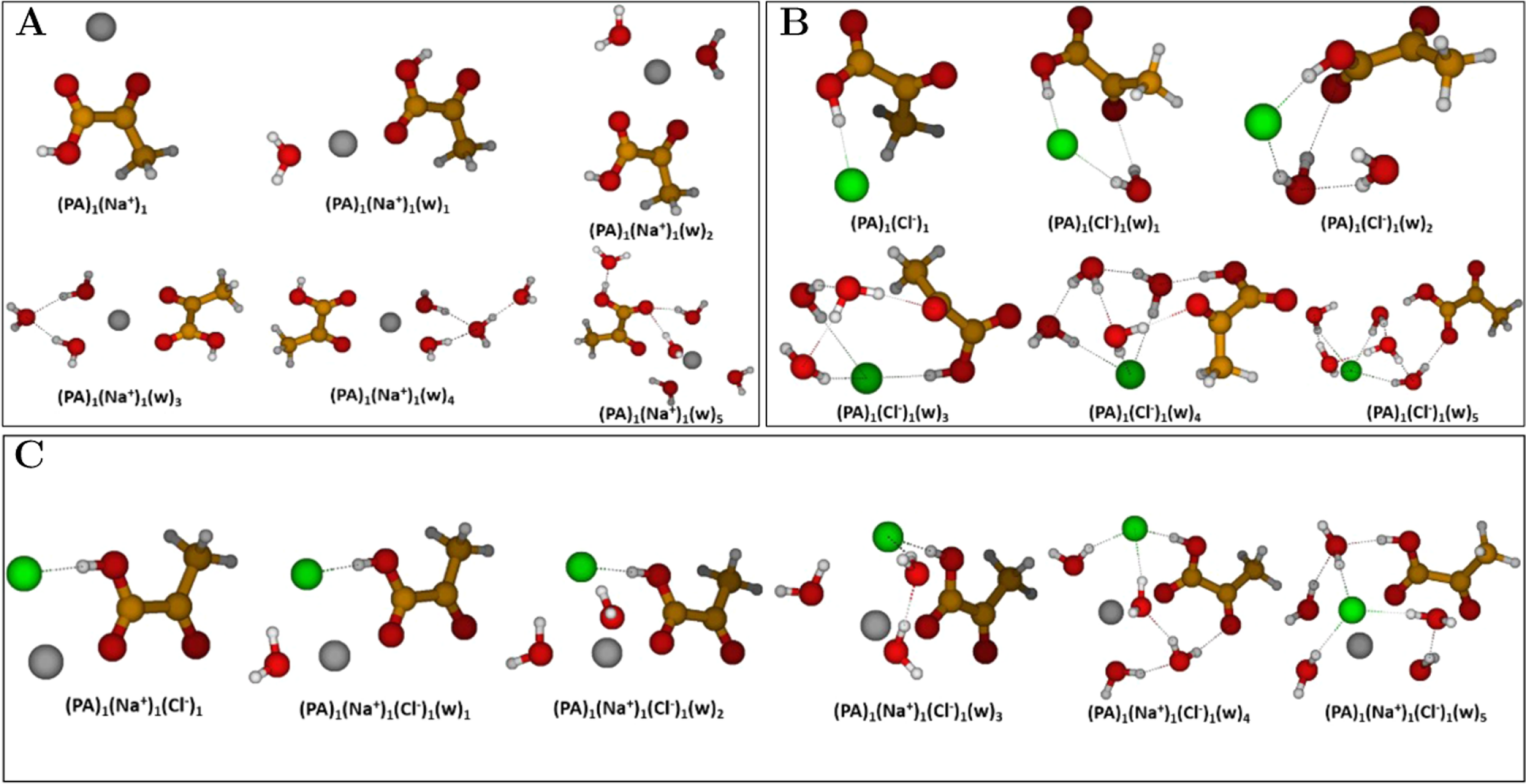
Lowest free energy clusters of PA, Na^+^, and Cl^–^with up to five water (w) molecules. (A) (PA)_1_(Na^+^)_1_(w)_0–5_; (B) (PA)_1_(Cl^–^)_1_(w)_0–5_; (C) (PA)_1_(Na^+^)_1_(Cl^–^)_1_(w)_0–5_. The geometries were obtained at the DLPNO–CCSD(T_0_)/aug-cc-pVTZ//ωB97X-D/6-31++G(d,p) level of theory, at 298.15 K and 1 atm using the quasi-harmonic approximation. Hydrogen atoms are white, carbon brown, oxygen red, sodium gray, and chlorine green.

We studied the (PA)_1_(Na^+^)_0–1_(Cl^–^)_0–1_(w)_0–5_ cluster systems to investigate the influence of ions on the PA-containing clusters. Figure 4 shows clusters with sodium ion, with chloride ion, and with both ions simultaneously.

Generally, we observe no proton transfers in these clusters. For the (PA)_1_(Na^+^)_1–5_ clusters in Figure 4A, PA prefers the **Ct** conformer. This can be ascribed to the bifurcated ion-dipole interactions between the adjacent carbonyl oxygens of PA and Na^+^. The water molecules are observed to reside on the other side of Na^+^ for the (PA)_1_(Na^+^)_1_(w)_1–4_ clusters thus building up a hydrogen-bonded water network solely interacting with the Na^+^ ion. However, for the (PA)_1_(Na^+^)_1_(w)_5_ cluster, we observe that the Na^+^ ion is ”solvated” by the water molecules, thus residing more remotely from the PA monomer.

Upon visual inspection of the (PA)_1_(Cl^–^)_1_(w)_0–5_ in Figure 4B, we observe a much broader variation in the preferred PA conformer across the hydrated clusters. PA prefers the **Cc** conformer in the (PA)_1_(Cl^–^)_1_ dimer cluster. For the (PA)_1_(Cl^–^)_1_(w)_1_ cluster, the C–C bond rotation between the carbonyl groups of PA has occurred and the **Tc** conformer is now preferred. The relative geometry between the carboxylic O–H group and the α-carbonyl group is not completely planar as it is in the ordinary **Tc** conformer, which can be ascribed to better steric access of Cl^–^ and the water molecule to engage in hydrogen bond interactions with the carboxylic O–H group and the α-carbonyl group, respectively. In the (PA)_1_(Cl^–^)_1_(w)_2_ cluster, the C–C bond has rotated further, and thus PA adopts the **Ct** form thereby interacting with Cl^–^ through the carboxylic O–H group and water through the carboxylic carbonyl group. For the (PA)_1_(Cl^–^)_1_(w)_3–4_ clusters, the C–C bond is rotated such that PA is effectively a superposition between the **Tc** and **Cc** conformers. Finally, in the (PA)_1_(Cl^–^)_1_(w)_5_ cluster, we observe the **Tt** conformer to be the preferred PA conformer. Here, we observe the water molecules to have formed a hydrogen bonding network encapsulating the Cl^–^ thus screening Cl^–^ from forming hydrogen bonds with the carboxylic O–H bond.

For the (PA)_1_(Na^+^)_1_(Cl^–^)_1_(w)_0–5_ clusters in Figure 4C, the **Ct** conformer is the preferred form across all clusters. The Cl^–^ ion engages in hydrogen bonding with the carboxylic O–H group in (PA)_1_(Na^+^)_1_(Cl^–^)_1_(w)_0–4_, while it is encapsulated by water and screened from interacting in (PA)_1_(Na^+^)_1_(Cl^–^)_1_(w)_5_. Generally across the clusters, we see that the Na^+^ ion and the Cl^–^ ion reside in close proximity to each other and form an ion-pair, which interacts with the carboxylic O–H group and the carbonyl group of the PA monomer. However, these interactions are mitigated when more water is added to the clusters. We also observe a general ion solvation point when reaching five waters in the various hydrated clusters where the ion is encapsulated and isolated from the PA monomer by the nascent water hydrogen bonding network.

Overall, introducing ions into the system highly perturbs the cluster geometries.

#### 3.1.4 Pyruvate-Containing Clusters

**Figure 5:**
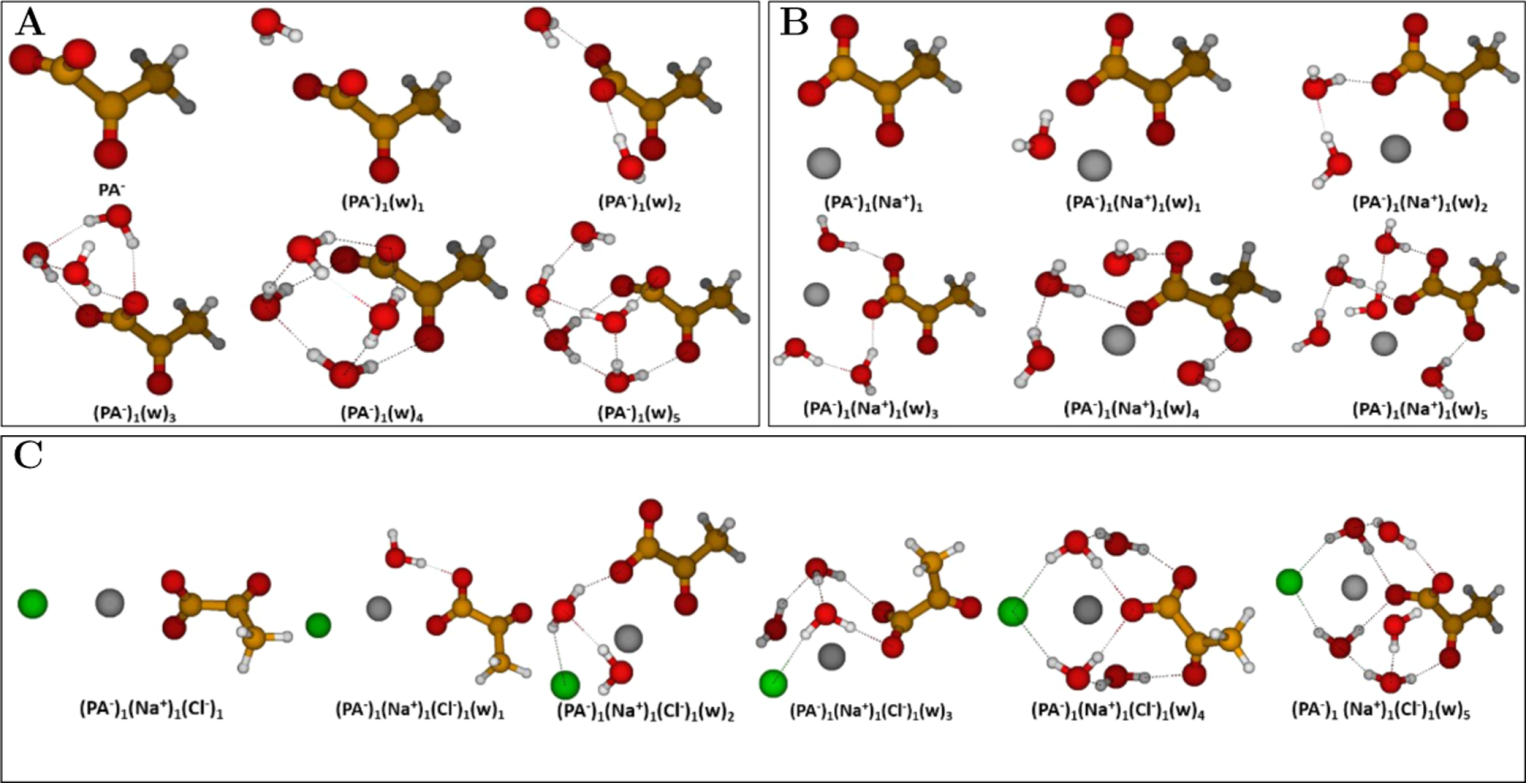
Lowest free energy conformers of pyruvate (PA^–^), Na^+^, and Cl^–^with up to five water (w) molecules: (A) (PA^–^)_1_(w)_0–5_; (B) (PA^–^)_1_(Na^+^)_1_(w)_0–5_; (C) (PA^–^)_1_(Na^+^)_1_(Cl^–^)_1_(w)_0–5_. The geometries were obtained at the DLPNO–CCSD(T_0_)/aug-cc-pVTZ//ωB97X-D/6-31++G(d,p) level of theory, at 298.15 K and 1 atm using the quasi-harmonic approximation. Hydrogen atoms are white, carbon brown, oxygen red, sodium gray, and chlorine green.

To study the conjugate base of PA, the pyruvate ion PA^–^, we investigated the (PA^–^)_1_(w)_0–5_ and (PA^–^)_1_(Na^+^)_1_(Cl^–^)_0–1_(w)_0–5_ cluster systems as presented in Figure 5.

In general, across all clusters in Figure 5, we observe no proton transfer events occurring from water to the PA^–^ monomer. For the (PA^–^)_1_(w)_0–5_ clusters in Figure 5A, we observed a nascent water hydrogen bonding network forming at the carboxylate terminus of PA^–^ analogous to our findings for the (PA)_1_(w)_0–5_ clusters above.

Upon comparison of the PA^–^ clusters with literature, we see that both the (PA^–^)_1_(w)_1_ and (PA^–^)_1_(w)_2_ clusters are consistent with the clusters obtained by Petersen-Sonn et al.^47^ obtained at CAM-B3LYP/aug-cc-pVTZ level of theory and the clusters obtained by Shemesh et al.^110^ at MP2/cc-pVDZ level of theory. As for the PA clusters examined previously, we observe discrepancies between our (PA^–^)_1_(w)_3–5_ cluster geometries and literature geometries due to the dispersed water structure in these and as the water structure in our geometries are well-ordered and mainly located at the carboxylate terminus of PA^–^.

Upon adding Na^+^ to the system in the (PA^–^)_1_(Na^+^)_1_(w)_0–5_ clusters of Figure 5B, we see that the Na^+^ ion preferably interacts with the two carbonyl groups instead of the carboxylate group. For the hydrated clusters, the Na^+^ ion is seen to bridge the water molecules and PA^–^. This is a similar interaction pattern as observed in the (PA)_1_(Na^+^)_1_(Cl^–^)_1_(w)_0–5_ clusters of Figure 4C.

For the (PA^–^)_1_(Na^+^)_1_(Cl^–^)_1_(w)_0–5_ clusters in Figure 5C, we generally observe Na^+^ to reside closer to the PA^–^ monomer where it engages in linear or bifurcated salt bridges with the carboxylate moiety of PA^–^. Cl^–^ is not observed to engage in hydrogen bonds with water or PA^–^ in the lower hydrated (PA^–^)_1_(Na^+^)_1_(Cl^–^)_1_ and (PA^–^)_1_(Na^+^)_1_(Cl^–^)_1_(w)_1_ clusters due to Coulomb repulsions. However, as more water is added, it becomes increasingly involved in the nascent water hydrogen bonding networks of the higher hydrated clusters. Na^+^ is, in this regard, becoming more encapsulated and solvated by the surrounding water, which we also observed for the (PA)_1_(Na^+^)_1_(Cl^–^)_1_(w)_0–5_ clusters as described above.

### 3.2 Cluster Binding Free Energies and Hydrate Distributions

After analyzing the cluster geometries, we progress to probe binding free energies and hydrate distributions. All calculations presented in this section were performed at the DLPNO–CCSD(T_0_)/aug-cc-pVTZ//ωB97X-D/6-31++G(d,p) level of theory, at 298.15 K and 1 atm using the quasi-harmonic approximation.

#### 3.2.1 Hydration of Organic Acids

**Figure 6:**
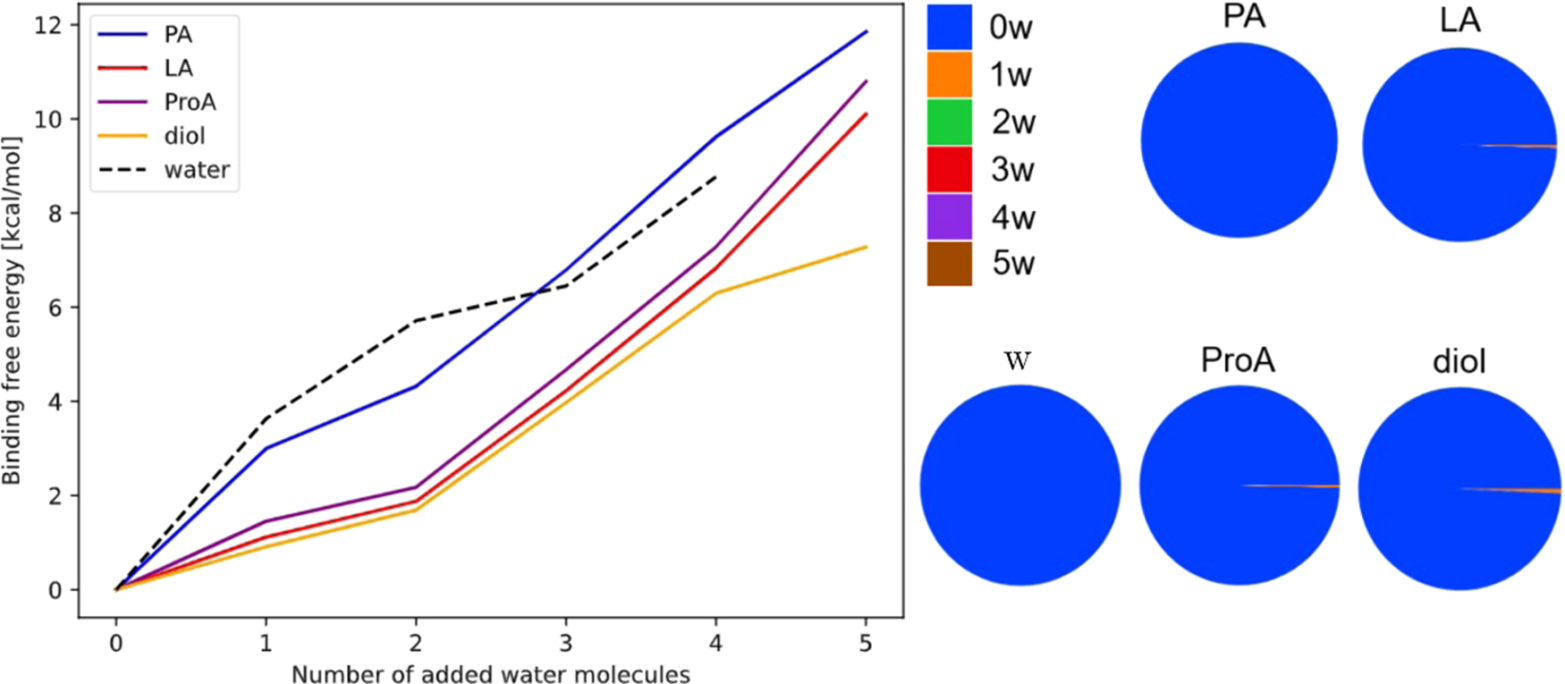
(Left): Binding free energies of (PA)_1_(w)_0–5_ (blue), (LA)_1_(w)_0–5_ (red), (ProA)_1_(w)_0–5_ (purple), and (diol)_1_(w)_0–5_ (orange) calculated at the DLPNO–CCSD(T_0_)/aug-cc-pVTZ//ωB97X-D/6-31++G(d,p) level of theory, at 298.15 K and 1 atm using the quasi-harmonic approximation. The binding free energies of pure water clusters are included as a black dashed line. (Right): Equilibrium hydrate distributions of the various organic acids and the water reference calculated at the DLPNO–CCSD(T_0_)/aug-cc-pVTZ//ωB97X-D/6-31++G(d,p) level of theory, at 298.15 K and 1 atm using the quasi-harmonic approximation and at 100% relative humidity. The hydrate distribution shows the fractional number of water molecules bound to the cluster (see eq 4).

The left panel of Figure 6 presents the gas-phase binding free energy profiles of PA and its various analogs: LA, ProA, and diol. The binding free energies are plotted as a function of “added water molecules”, such that each point has the same total number of molecules in the cluster. Hence, zero is the monomer of either an organic acid or a water molecule.

All binding free energies are positive and increase with the number of water molecules in the cluster. This entails that the clusters become increasingly more thermodynamically unfavorable and thus increasingly unstable in the gas phase when adding more water molecules. This is most pronounced for the (PA)_1_(w)_0–5_ clusters as they are observed to be close to or even higher in free energy compared to the pure gas-phase water clusters. The higher free energy is caused by the internal H-bond present in the PA monomer. Hence, to form clusters with water, PA needs to break the internal H-bond, which thereby does not lead to a gain in free energy. This trend has also been observed for other organic acids such as pinic acid, 3-methyl-1,2,3-butanetricarboxylic acid (mbtca), and 2-oxohexanediperoxy acid^111^

The first hydration steps of LA, ProA, and diol is more favorable compared to PA. In addition, the binding free energies are very similar for the addition of the first four water molecules. However, the addition of the fifth water molecule is significantly more favorable for the diol compared to LA and ProA. This can be rationalized from the geometries in Figure 3, where the diol is interacting with all five water molecules in the extended hydrogen bonding network, while LA and ProA primarily interact with four water molecules and have the fifth water molecule only interacting with other water molecules and not the acids.

The right panel of Figure 6 presents the hydrate distributions of the organic acids. All the organic acids are observed to be nonhydrated in the gas phase. Hydrate distributions obtained at 273.15 K and 258.15 K and at 50% relative humidity do not change this conclusion (see Supporting Information).

#### 3.2.2 Pyruvic Acid-Containing Clusters

**Figure 7:**
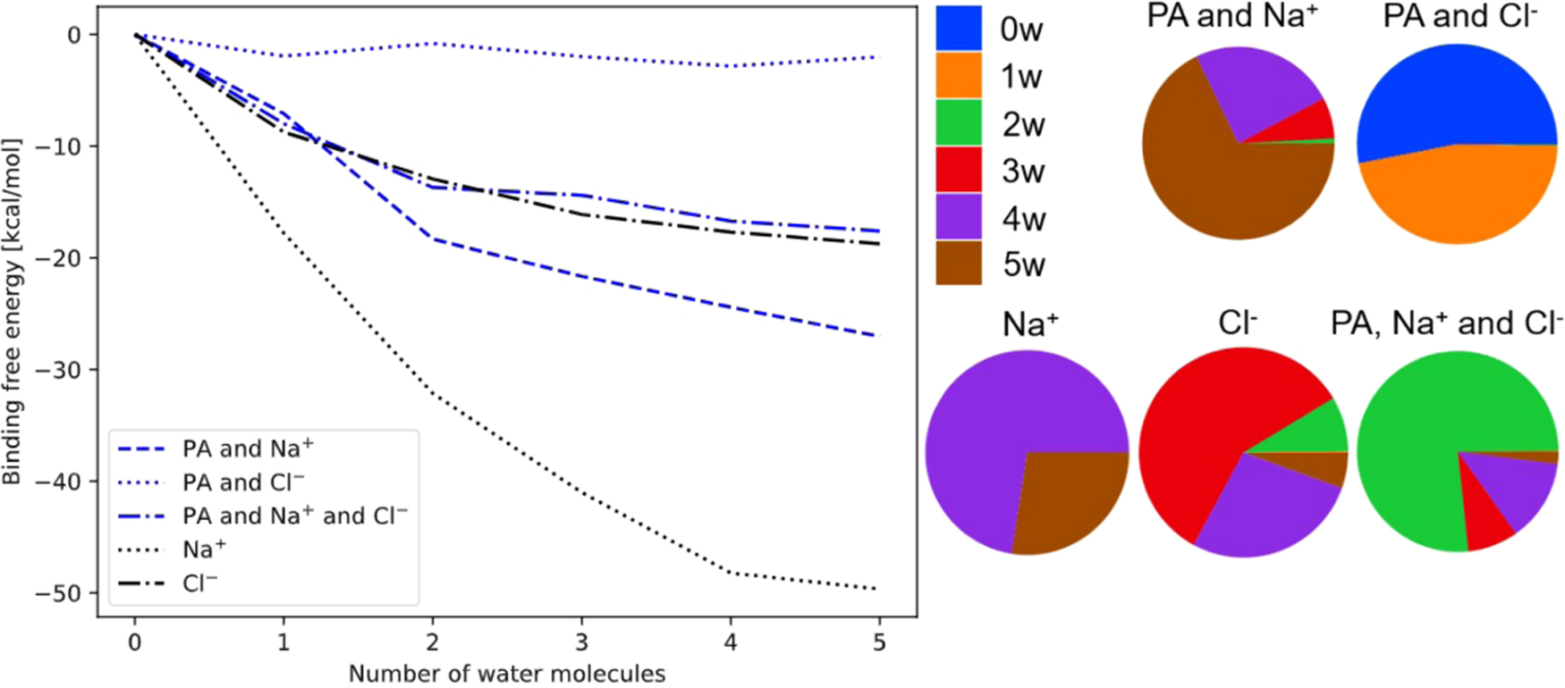
(Left): Normalized binding free energies with respect to the dry clusters of (PA)_1_(Na^+^)_1_(w)_0–5_ (blue dashed), (PA)_1_(Cl^–^)_1_(w)_0–5_ (blue dotted), and (PA)_1_(Na^+^)_1_(Cl^–^)_1_(w)_0–5_ (blue dash-dotted) calculated at the DLPNO–CCSD(T_0_)/aug-cc-pVTZ//ωB97X-D/6-31++G(d,p) level of theory, at 298.15 K and 1 atm using the quasi-harmonic approximation. The binding free energies of the reference (Na^+^)_1_(w)_0–5_ and (Cl^–^)_1_(w)_0–5_ clusters are included as a black dotted and a black dash-dotted line, respectively. (Right): Equilibrium hydrate distributions of the various clusters calculated at the DLPNO–CCSD(T_0_)/aug-cc-pVTZ//ωB97X-D/6-31++G(d,p) level of theory, at 298.15 K and 1 atm using the quasi-harmonic approximation and at 100% relative humidity.

The binding free energy profiles of the PA clusters containing Na^+^ and Cl^–^ ions were investigated. The left panel of Figure 7 presents the gas-phase binding free energies of PA with Na^+^ and Cl^–^. In contrast to the positive binding free energies of the hydrated clusters containing PA, LA, ProA, and diol as discussed above, we generally observe the hydrated PA clusters containing ions to be thermodynamically favorable. Especially the (PA)_1_(Na^+^)_1_(w)_0–5_ and the (PA)_1_(Na^+^)_1_(Cl^–^)_1_(w)_0–5_ clusters are seen to decrease markedly at smaller water content and then flatten out at larger water content. This indicates that the stabilizing effect is diminishing with increasing water content and is a usual phenomenon observed in the microhydration of ions. On the other hand, the (PA)_1_(Cl^–^)_1_(w)_0–5_ clusters are observed to be relatively constant, just below zero in normalized binding free energy, which indicates that Cl^–^ is not able to thermodynamically stabilize the hydrated clusters in the gas phase as favorably as the hydrated clusters containing Na^+^. In general, the hydration of the pyruvic acid-containing clusters in the presence of ions is negative and thus the ions may compensate for the lack of binding observed in the pure PA–water clusters. In the case of (PA)_1_(Cl^–^)_1_(w)_0–5_, the binding free energy is nearly zero as the negative free energy of the (Cl^–^)_1_(w)_0–5_ clusters cancel with the positive free energy of the (PA)_1_(w)_0–5_ clusters. In the case of the (PA)_1_(Na^+^)_1_(w)_0–5_ clusters, the presence of Na^+^ still leads to a low free energy. It is surprising that there is such a huge difference between the Na^+^/Cl^–^ case and especially the NaCl cases. For instance, it is noteworthy that the net-neutral (PA)_1_(Na^+^)_1_(Cl^–^)_1_(w)_0–5_ case is more hydrated than the net-charged (PA)_1_(Cl^–^)_1_(w)_0–5_ case. This contradicts the simple classical charge-dipole interaction physics.

To investigate whether PA is capable of displacing water molecules from the solvation shell of the ions, we calculated the following two types of water displacement reactions
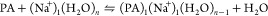
5
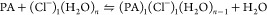
6We find that the free energies for the water displacement reactions are negative for both ions and at all hydration levels (see Figure S2 in the Supporting Information). The water displacement reactions are most favorable at low hydration levels with a free energy of roughly −12 kcal/mol at *n* = 1, increasing to around −5 to −6 kcal/mol at *n* = 5. This illustrates that PA is capable of displacing water molecules from the hydration shell of the ions.

The right panel of Figure 7 presents the hydrate distributions of the pyruvic acid-containing clusters. Generally, we observe the highly hydrated clusters to be favored when the binding free energy is very negative and we observe the (PA)_1_(Na^+^)_1_(w)_0–5_ clusters to favor hydration the most. Similar conclusion is reached when studying the hydrate distributions obtained at 273.15 K and 258.15 K and at 50% relative humidity (available in the Supporting Information).

When comparing the binding free energy analysis and the hydrate distributions with the cluster geometry analysis in the previous section, it is clear that Na^+^ ions stabilize the PA clusters the most and the stabilization is less pronounced for the Cl^–^ ion. Furthermore, a significant degree of solvation of the Na^+^ ion by water in the gas phase is also thermodynamically favorable for stabilizing the clusters.

#### 3.2.3 Pyruvate-Containing Clusters

**Figure 8:**
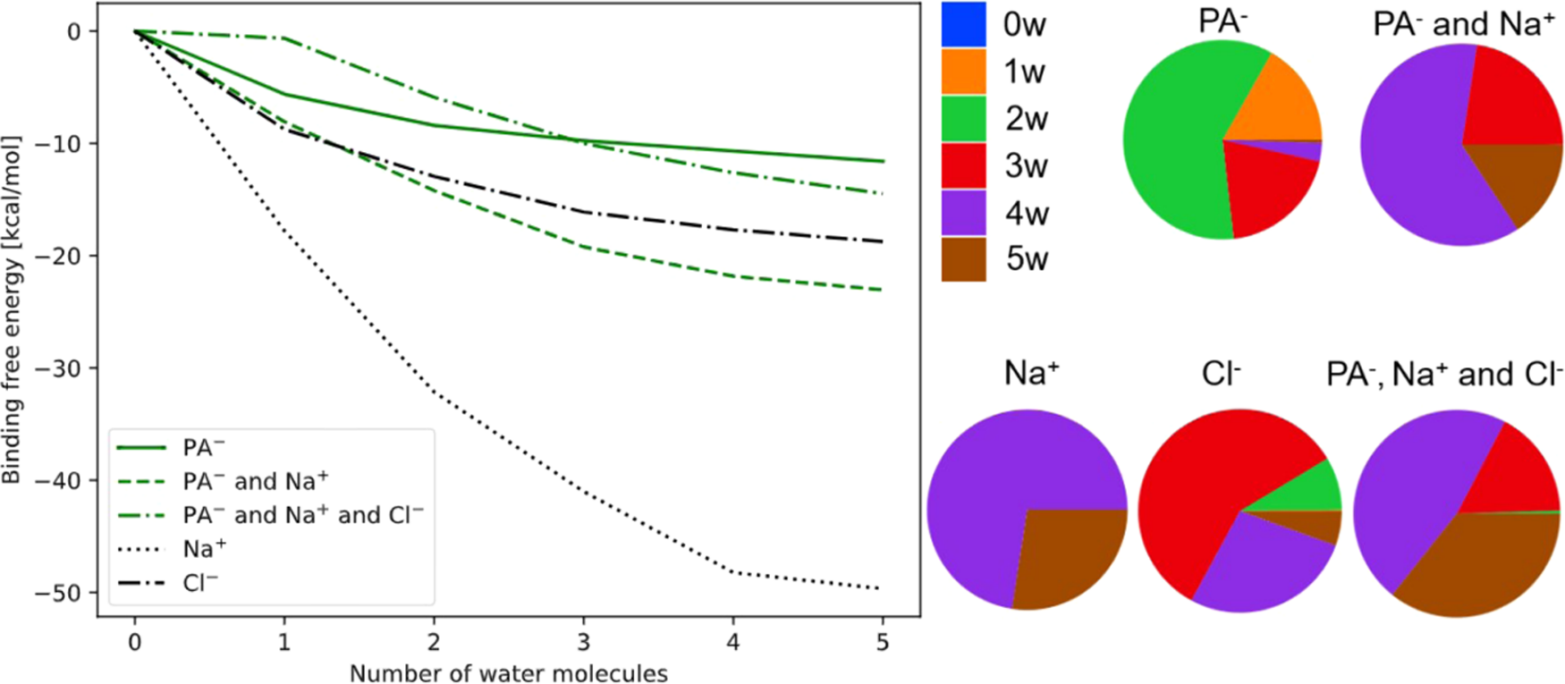
(Left): Normalized binding free energies with respect to the dry clusters of (PA^–^)_1_(w)_0–5_, (PA^–^)_1_(Na^+^)_1_(w)_0–5_, and (PA^–^)_1_(Na^+^)_1_(Cl^–^)_1_(w)_0–5_ calculated at the DLPNO–CCSD(T_0_)/aug-cc-pVTZ//ωB97X-D/6-31++G(d,p) level of theory, at 298.15 K and 1 atm using the quasi-harmonic approximation. (Right): Equilibrium hydrate distributions of the various clusters calculated at the DLPNO–CCSD(T_0_)/aug-cc-pVTZ//ωB97X-D/6-31++G(d,p) level of theory, at 298.15 K and 1 atm using the quasi-harmonic approximation and at 100% relative humidity.

Deprotonation of PA leading to PA^–^ could lead to more stable clusters. The left panel of Figure 8 presents the binding free energies normalized to the dry clusters of PA^–^ with Na^+^ and Cl^–^. In general, the hydration profiles are similar to the PA-containing clusters described above. We observe the energies of the (PA^–^)_1_(w)_0–5_, (PA^–^)_1_(Na^+^)_1_(w)_0–5_ and (PA^–^)_1_(Na^+^)_1_(Cl^–^)_1_(w)_0–5_ to decrease with increasing water content. This leads to negative binding free energies in all cases. Hence, pyruvate hydration is favorable and seems to be enhanced by other ions. Again, it is noteworthy that the net-neutral (PA^–^)_1_(Na^+^)_1_(w)_0–5_ case is more hydrated than the net-charged (PA^–^)_1_(Na^+^)_1_(Cl^–^)_1_(w)_0–5_ case.

The right panel of Figure 8 presents the hydrate distributions of the pyruvate-containing clusters. In all cases, the clusters are highly hydrated with 2–5 water molecules. The (PA^–^)_1_(Na^+^)_1_(Cl^–^)_1_(w)_0–5_ clusters are found to be slightly more hydrated than the corresponding (PA^–^)_1_(Na^+^)_1_(w)_0–5_ clusters. This could indicate that Cl^–^ exhibits a synergistic effect with Na^+^ in thermodynamically stabilizing the higher hydrated clusters. Hydrate distributions obtained at 273.15 K and 258.15 K and at 50% relative humidity show similar trends (see Supporting Information).

Overall, we find that ions are very important for the interaction between the PA monomer and water as well as between the PA^–^ monomer and water addressed here. Hence, for organic acids to efficiently partition into the particle phase some surface ions should most likely be present.

### 3.3 Harmonic IR Absorption

Gas-phase measurements of hydrated clusters are extremely difficult to carry out. One promising technique to identify weakly bound clusters is to probe the H-shift of the O–H stretch upon cluster formation. This methodology has been applied to detect clusters such as the gas-phase PA monomer,^60^ the gas-phase dimethylamine monomer,^112^ and the gas-phase methanol–dimethylamine dimer^113^ where the N–H stretch is contributing to cluster formation. However, to the best of our knowledge, there are no previous studies on the IR absorptions of hydrated PA clusters in the gas phase. Here we provide such analysis to potentially guide future experiments. The IR spectra presented in this section were obtained at ωB97X-D/6-31++G(d,p) level of theory. The O–H stretch is known to exhibit anharmonic behavior, which is neglected in the current study. However, the change in the frequency due to anharmonicity should be relatively constant across all the O–H stretches, simply leading to a shift in the total spectrum. We stress that the choice of method is qualitatively sufficient for the following analysis, i.e., the peaks in the IR spectra are likely shifted compared to experimental values, but the overall relative peak positions should be accurate.

#### 3.3.1 IR Absorption of the Hydrated Pyruvic Acid Clusters

**Figure 9:**
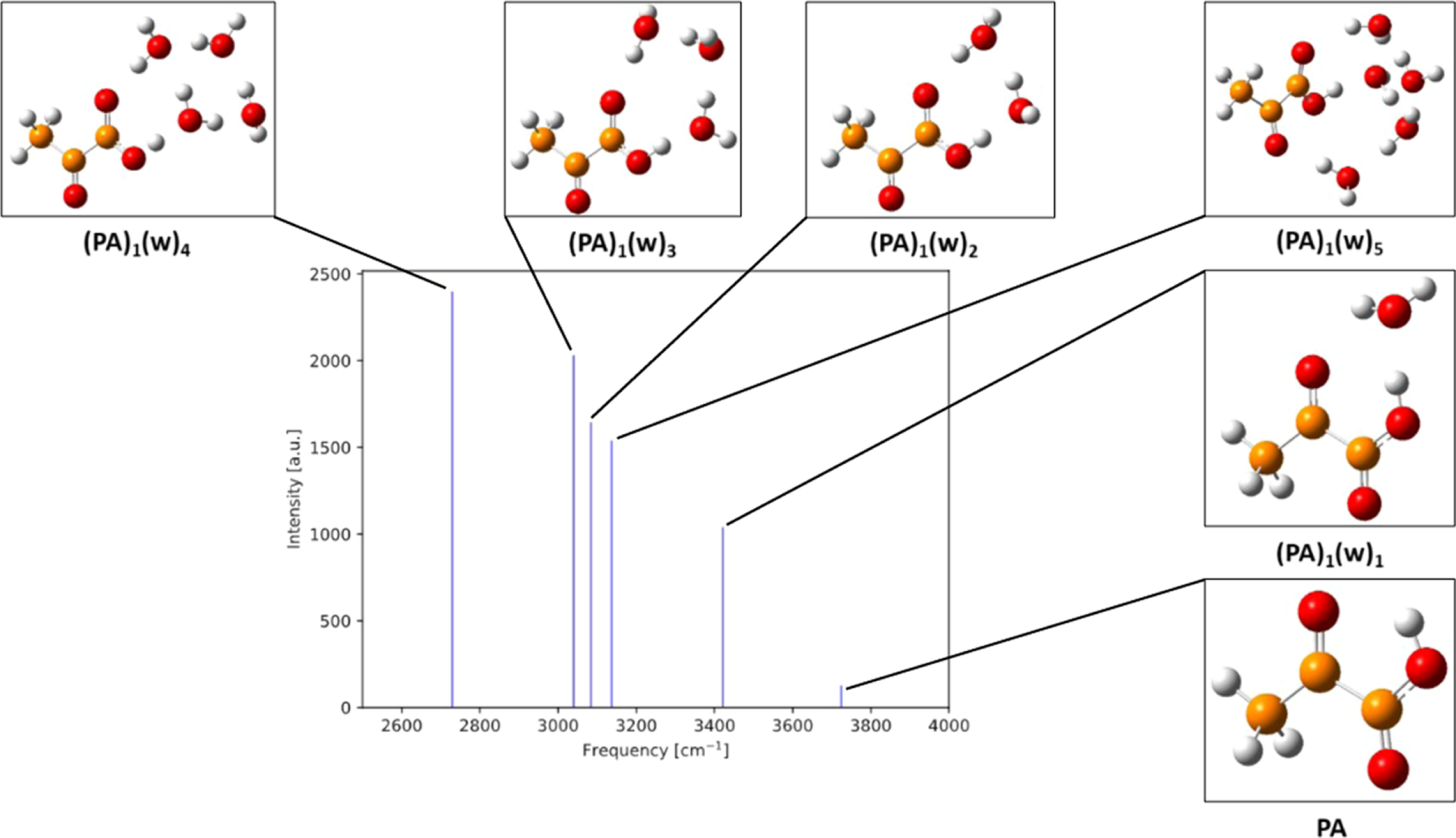
IR intensities as a function of IR O–H frequencies corresponding to the carboxylic O–H stretching mode of PA for (PA)_1_(w)_0–5_ calculated at the ωB97X-D/6-31++G(d,p) level of theory. Hydrogen atoms are white, carbon brown, and oxygen red.

Figure 9 presents the IR O–H frequencies and intensities of the hydrated gas-phase PA clusters with corresponding cluster geometries. In general, we see a trend of decreasing O–H frequency (redshift) and increasing intensity with increasing cluster water content. As the PA monomer is found not to be hydrated in the gas phase, a strong redshift from 3725 cm^–1^ in an experiment could indicate that the PA monomer is interacting with one or more water molecules. This stretching frequency for the gas-phase PA monomer is also in fairly good agreement with previously reported experimental work.^60^

#### 3.3.2 IR Absorption of the Clusters Containing Pyruvic Acid and Na^+^

**Figure 10:**
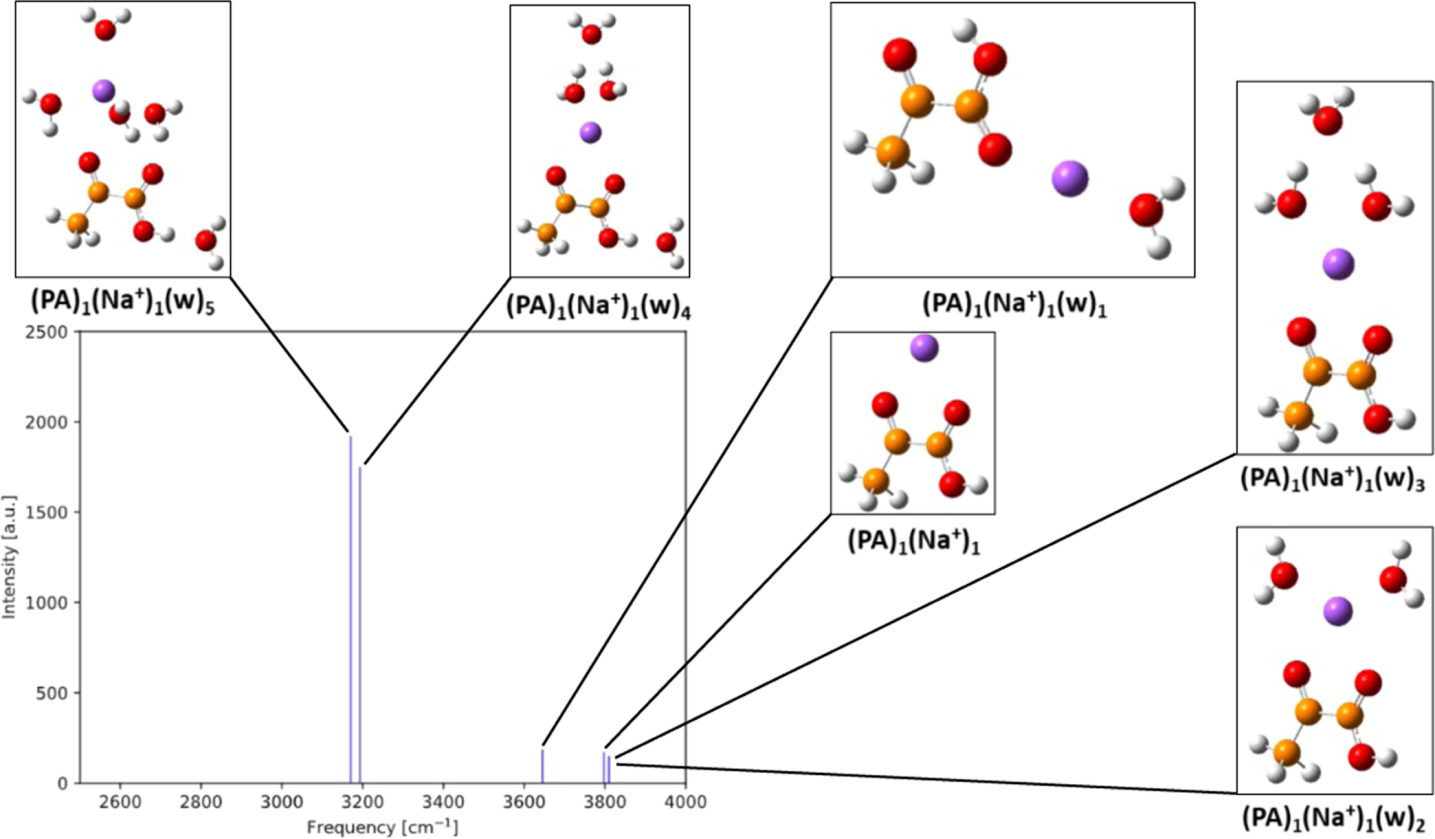
IR intensities as a function of IR O–H frequencies for (PA)_1_(Na^+^)_1_(w)_0–5_ calculated at the ωB97X-D/6-31++G(d,p) level of theory. Hydrogen atoms are white, carbon brown, oxygen red, and sodium purple.

Figure 10 presents the IR O–H frequencies and intensities of the hydrated gas-phase PA clusters with Na^+^. In contrast to the hydrated PA clusters described above, we generally observe a more mixed pattern in O–H frequencies with respect to cluster water content. The difference in intensities in the spectrum can be attributed to the presence of water at the O–H bond, as seen in the (PA)_1_(Na^+^)_1_(w)_4_ and (PA)_1_(Na^+^)_1_(w)_5_ clusters, with redshifts of 532 cm^–1^ and 555 cm^–1^ compared to the PA monomer, respectively. The remaining clusters do not have water present at the O–H bond, which leads to an O–H stretching frequency similar to the isolated PA monomer.

#### 3.3.3 IR Absorptions of the Clusters Containing Pyruvic Acid and Cl^–^

**Figure 11:**
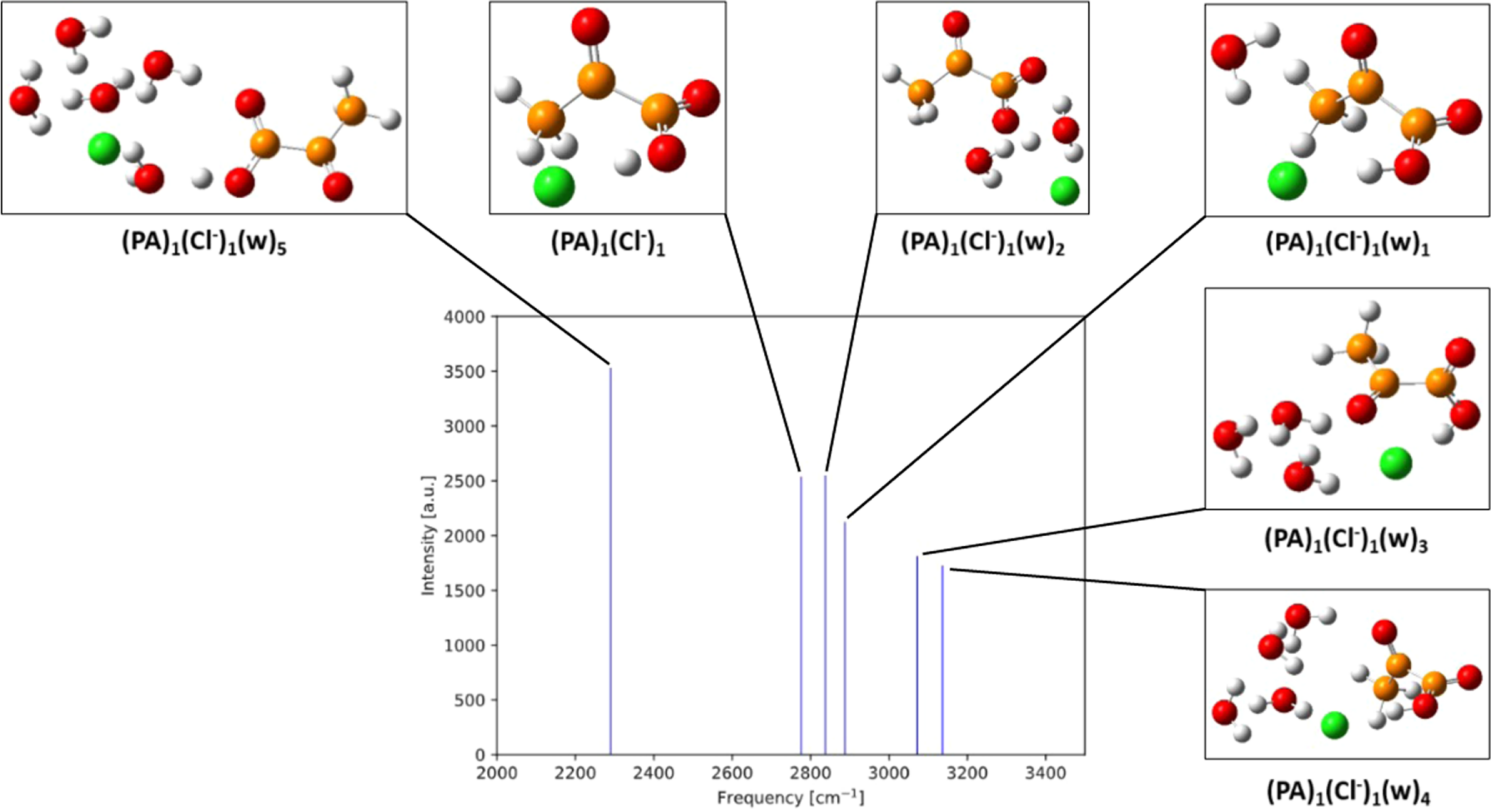
IR intensities as a function of IR O–H frequencies for (PA)_1_(Cl^–^)_1_(w)_0–5_ calculated at the ωB97X-D/6-31++G(d,p) level of theory. Hydrogen atoms are white, carbon brown, oxygen red, and chlorine green.

Figure 11 shows the IR O–H frequencies and intensities of the hydrated gas-phase PA clusters with Cl^–^. We generally observe the O–H frequency pattern with respect to water content to be even more mixed here than for the (PA)_1_(Na^+^)_1_(w)_0–5_ clusters. The O–H stretching frequency is significantly redshifted (by up to 1435 cm^–1^) compared to the PA monomer in all cases. This is caused by the strong interaction between the Cl^–^ ion and the O–H group. Hence, a very strong redshift in the IR-spectrum could indicate the presence of a tight ion-pair with chloride ion.

#### 3.3.4 IR Absorption of the Clusters Containing Pyruvic Acid, Na^+^, and Cl^–^

**Figure 12:**
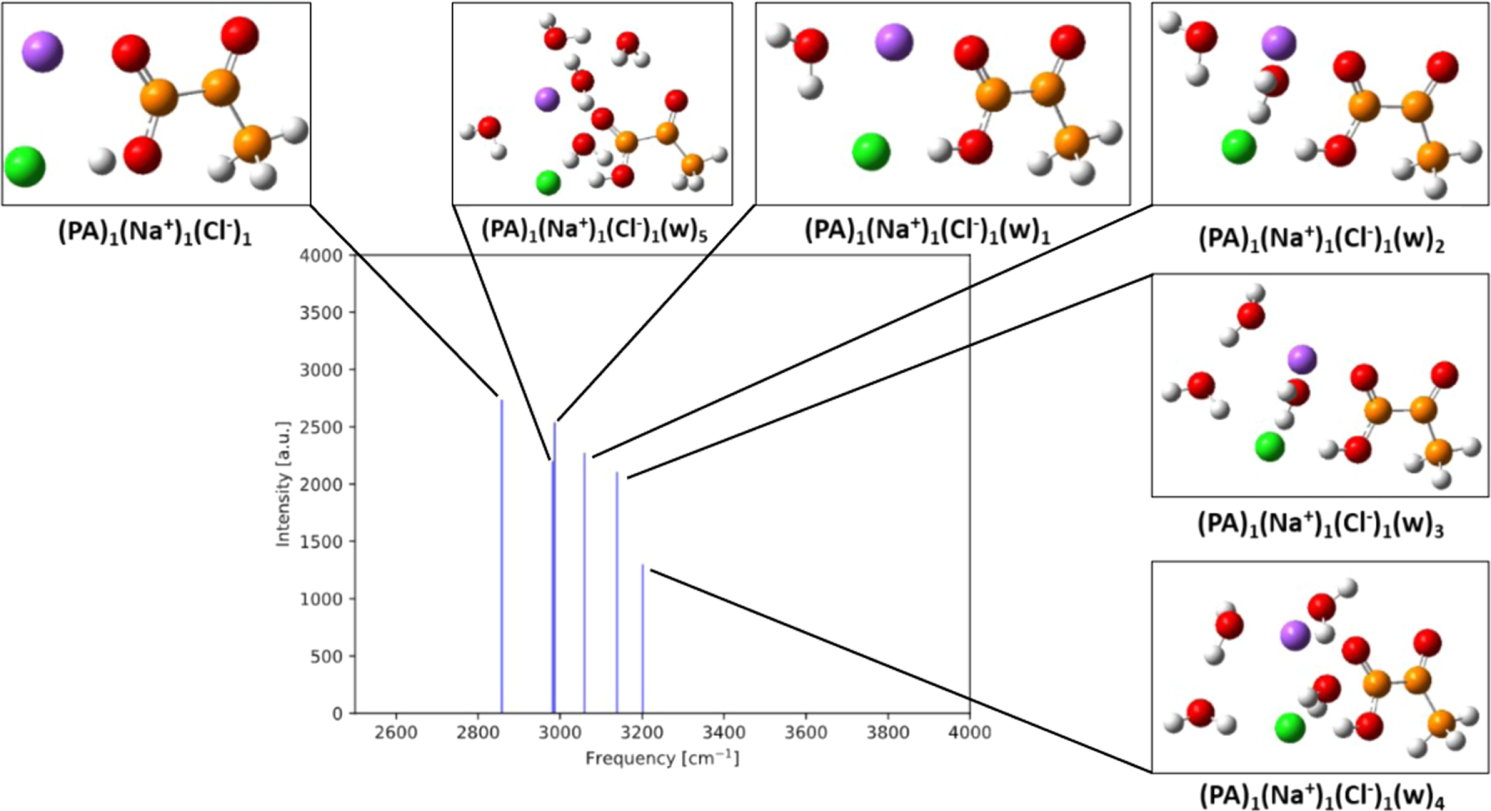
IR intensities as a function of IR O–H frequencies for (PA)_1_(Na^+^)_1_(Cl^–^)_1_(w)_0–5_ calculated at the ωB97X-D/6-31++G(d,p) level of theory. Hydrogen atoms are white, carbon brown, oxygen red, sodium purple, and chlorine green.

Figure 12 shows the IR intensities as a function of IR O–H frequencies of the hydrated gas-phase clusters with Na^+^ and Cl^–^. All the clusters are redshifted by up to 867 cm^–1^ compared to the PA monomer. In addition, we observe the O–H frequencies to increase with increasing water content while the intensities decrease. This is in contrast to the hydrated PA clusters with no ions present.

To summarize the above IR analyses of the various systems, we can corroborate the correlation between decreasing O–H frequency and increasing intensity with increasing water content of the clusters, which is gradually reversed when adding ions to the system. Finally, we see a propensity toward redshifting of the O–H vibrational frequencies when adding more ions to the hydrated PA clusters even though the O–H frequencies in the (PA)_1_(Na^+^)_1_(w)_0–5_ clusters appear consistently above 3000 cm^–1^.

## 4 Conclusions

Here we have studied the clustering of pyruvic acid (PA) and structural analogs of PA, water, and Na^+^/Cl^–^ ions. As a first approximation, we focus on the gas-phase PA accommodation and interaction with the local environment of an aerosol surface, which is here only represented by a small cluster of molecules. In our study of the cluster geometries of the reference systems comprised of pure water, hydrated Na^+^, and hydrated Cl^–^, we find that all are in full agreement with literature. This illustrates the applicability of the applied computational approach.

For the cluster geometries of hydrated pyruvate, PA^–^, and organic acids (including PA), we show that for all clusters containing one to four water molecules, a complete conservation of the water network structure with respect to the pure water clusters is preserved. However, upon introduction of ions into the pyruvic acid-containing clusters and pyruvate-containing clusters, we show that the water structure of the clusters are perturbed significantly compared to the organic acid clusters without ions and the reference systems.

In our investigation of the cluster binding free energies and hydrate distributions, we find that the organic acids PA, LA, ProA, and diol are not hydrated in the gas phase. On the other hand, we find that the pyruvic-acid containing clusters and the pyruvate-containing clusters with ions present are significantly more thermodynamically stabilized in the gas phase with Na^+^ contributing the most to this stabilization. Intriguingly, we observe that the net-neutral clusters containing ion-pairs can be more hydrated than net-charged clusters. This finding contradicts the classical view on charge-dipole interactions.

This suggests that for organic acids, to efficiently transition from the gas phase to the particle phase, some interfacial surface ions should possibly be present to facilitate the process.

As a part of the IR absorption studies of the various pyruvic acid-containing clusters, we find a correlation between decreasing O–H frequency and increasing intensity when more water is added to the clusters. Furthermore, we observe a correlation between an increasing redshift of the O–H frequencies upon addition of ions to the clusters indicating strong interactions between the PA monomer and the surrounding water and ions.

In the following work to this study, we will investigate the same systems in the aqueous phase and at aerosol surfaces to probe the cluster geometries and stabilities when changing the chemical environment. In addition, it could be interesting to further study the interconversion between the keto and diol forms of PA and how the local environment potentially shifts this equilibrium. It could also be interesting to investigate how the local environment potentially affects the photolysis of PA. Thereby we can fully unravel the mechanisms, which leads to favorable partitioning of organic acids into aerosol particles, where they can contribute to atmospheric particle growth.

## Supporting Information Available

Assessment of pyruvic acid conformers; assessment of pyruvic acid interaction energies; initial benchmark results; cluster binding free energies and hydrate distributions at various temperatures (PDF)

## References

[ref1] Myhre C. E. L.; Nielsen C. J. Optical Properties in the UV and Visible Spectral Region of Organic Acids Relevant to Tropospheric Aerosols. Atmos. Chem. Phys. 2004, 4, 1759–1769. 10.5194/acp-4-1759-2004.

[ref2] Eger P. G.; Schuladen J.; Sobanski N.; Fischer H.; Karu E.; Williams J.; Riva M.; Zha Q.; Ehn M.; Quéléver L. L. J.; Schallhart S.; Lelieveld J.; Crowley J. N. Pyruvic Acid in the Boreal Forest: Gas-Phase Mixing Ratios and Impact on Radical Chemistry. Atmos. Chem. Phys. 2020, 20, 3697–3711. 10.5194/acp-20-3697-2020.

[ref3] Kawamura K.; Tachibana E.; Okuzawa K.; Aggarwal S. G.; Kanaya Y.; Wang Z. F. High Abundances of Water-Soluble Dicarboxylic Acids, Ketocarboxylic Acids and α-Dicarbonyls in the Mountaintop Aerosols over the North China Plain during Wheat Burning Season. Atmos. Chem. Phys. 2013, 13, 8285–8302. 10.5194/acp-13-8285-2013.

[ref4] Kawamura K.; Bikkina S. A Review of Dicarboxylic Acids and Related Compounds in Atmospheric Aerosols: Molecular Distributions, Sources and Transformation. Atmos. Res. 2016, 170, 140–160. 10.1016/j.atmosres.2015.11.018.

[ref5] Kawamura K.; Yasui O. Diurnal Changes in the Distribution of Dicarboxylic Acids, Ketocarboxylic Acids and Dicarbonyls in the Urban Tokyo Atmosphere. Atmos. Environ. 2005, 39, 1945–1960. 10.1016/j.atmosenv.2004.12.014.

[ref6] Ho K. F.; Lee S. C.; Cao J. J.; Kawamura K.; Watanabe T.; Cheng Y.; Chow J. C. Dicarboxylic Acids, Ketocarboxylic Acids and Dicarbonyls in the Urban Roadside Area of Hong Kong. Atmos. Environ. 2006, 40, 3030–3040. 10.1016/j.atmosenv.2005.11.069.

[ref7] Ho K. F.; Cao J. J.; Lee S. C.; Kawamura K.; Zhang R. J.; Chow J. C.; Watson J. G. Dicarboxylic Acids, Ketocarboxylic Acids, and Dicarbonyls in the Urban Atmosphere of China. J. Geophys. Res.:Atmos. 2007, 112, S27-1–S27-12. 10.1029/2006JD008011.

[ref8] Ho K. F.; Lee S. C.; Ho S. S. H.; Kawamura K.; Tachibana E.; Cheng Y.; Zhu T. Dicarboxylic Acids, Ketocarboxylic Acids, α-Dicarbonyls, Fatty Acids, and Benzoic Acid in Urban Aerosols Collected during the 2006 Campaign of Air Quality Research in Beijing (CAREBeijing-2006). J. Geophys. Res.: Atmos. 2010, 115, 3111–3123. 10.1029/2009jd013304.

[ref9] Jung J.; Tsatsral B.; Kim Y. J.; Kawamura K. Organic and Inorganic Aerosol Compositions in Ulaanbaatar, Mongolia, during the Cold Winter of 2007 to 2008: Dicarboxylic Acids, Ketocarboxylic Acids, and α-Dicarbonyls. J. Geophys. Res.: Atmos. 2010, 115, D22203 10.1029/2010JD014339.

[ref10] Pavuluri C. M.; Kawamura K.; Swaminathan T. Water-Soluble Organic Carbon, Dicarboxylic Acids, Ketoacids, and α-Dicarbonyls in the Tropical Indian Aerosols. J. Geophys. Res.: Atmos. 2010, 115, D11302 10.1029/2009jd012661.

[ref11] Kawamura K. Identification of C2-C10 ω-oxocarboxylic acids, pyruvic acid, and C2-C3 α-Dicarbonyls in Wet Precipitation and Aerosol Samples by Capillary GC and GC/MS. Anal. Chem. 1993, 65, 3505–3511. 10.1021/ac00071a030.

[ref12] Wang H.; Kawamura K.; Yamazaki K. Water-Soluble Dicarboxylic Acids, Ketoacids and Dicarbonyls in the Atmospheric Aerosols over the Southern Ocean and Western Pacific Ocean. J. Atmos. Chem. 2006, 53, 43–61. 10.1007/s10874-006-1479-4.

[ref13] Fu P.; Kawamura K.; Usukura K.; Miura K. Dicarboxylic Acids, Ketocarboxylic Acids and Glyoxal in the Marine Aerosols Collected during a Round-The-World Cruise. Mar. Chem. 2013, 148, 22–32. 10.1016/j.marchem.2012.11.002.

[ref14] Kawamura K.; Kasukabe H.; Barrie L. A. Secondary Formation of Water-Soluble Organic Acids and α-Dicarbonyls and Their Contributions to Total Carbon and Water-Soluble Organic Carbon: Photochemical Aging of Organic Aerosols in the Arctic Spring. J. Geophys. Res.: Atmos. 2010, 115, D21306 10.1029/2010JD014299.

[ref15] Kundu S.; Kawamura K.; Andreae T. W.; Hoffer A.; Andreae M. O. Molecular Distributions of Dicarboxylic Acids, Ketocarboxylic Acids and α-Dicarbonyls in Biomass Burning Aerosols: Implications for Photochemical Production and Degradation in Smoke Layers. Atmos. Chem. Phys. 2010, 10, 2209–2225. 10.5194/acp-10-2209-2010.

[ref16] Griffith E. C.; Carpenter B. K.; Shoemaker R. K.; Vaida V. Photochemistry of Aqueous Pyruvic Acid. Proc. Natl. Acad. Sci. U.S.A. 2013, 110, 11714–11719. 10.1073/pnas.1303206110. 23821751 PMC3718102

[ref17] Harris A. E. R.; Ervens B.; Shoemaker R. K.; Kroll J. A.; Rapf R. J.; Griffith E. C.; Monod A.; Vaida V. Photochemical Kinetics of Pyruvic Acid in Aqueous Solution. J. Phys. Chem. A 2014, 118, 8505–8516. 10.1021/jp502186q. 24725260

[ref18] Eugene A. J.; Guzman M. I. Reactivity of Ketyl and Acetyl Radicals from Direct Solar Actinic Photolysis of Aqueous Pyruvic Acid. J. Phys. Chem. A 2017, 121, 2924–2935. 10.1021/acs.jpca.6b11916. 28362101

[ref19] Rapf R. J.; Perkins R. J.; Carpenter B. K.; Vaida V. Mechanistic Description of Photochemical Oligomer Formation from Aqueous Pyruvic Acid. J. Phys. Chem. A 2017, 121, 4272–4282. 10.1021/acs.jpca.7b03310. 28510434

[ref20] Rapf R. J.; Dooley M. R.; Kappes K.; Perkins R. J.; Vaida V. pH Dependence of the Aqueous Photochemistry of α-Keto Acids. J. Phys. Chem. A 2017, 121, 8368–8379. 10.1021/acs.jpca.7b08192. 29032688

[ref21] Maroń M. K.; Takahashi K.; Shoemaker R. K.; Vaida V. Hydration of Pyruvic Acid to its Geminal-Diol, 2, 2-Dihydroxypropanoic Acid, in a Water-Restricted Environment. Chem. Phys. Lett. 2011, 513, 184–190. 10.1016/j.cplett.2011.07.090.

[ref22] Guzmán M. I.; Colussi A. J.; Hoffmann M. R. Photoinduced Oligomerization of Aqueous Pyruvic Acid. J. Phys. Chem. A 2006, 110, 3619–3626. 10.1021/jp056097z. 16526643

[ref23] Ault A. P. Aerosol Acidity: Novel Measurements and Implications for Atmospheric Chemistry. Acc. Chem. Res. 2020, 53, 1703–1714. 10.1021/acs.accounts.0c00303. 32786333

[ref24] Harris A. E. R.; Pajunoja A.; Cazaunau M.; Gratien A.; Pangui E.; Monod A.; Griffith E. C.; Virtanen A.; Doussin J.-F.; Vaida V. Multiphase Photochemistry of Pyruvic Acid under Atmospheric Conditions. J. Phys. Chem. A 2017, 121, 3327–3339. 10.1021/acs.jpca.7b01107. 28388049

[ref25] Kappes K. J.; Deal A. M.; Jespersen M. F.; Blair S. L.; Doussin J.-F.; Cazaunau M.; Pangui E.; Hopper B. N.; Johnson M. S.; Vaida V. Chemistry and Photochemistry of Pyruvic Acid at the Air–Water Interface. J. Phys. Chem. A 2021, 125, 1036–1049. 10.1021/acs.jpca.0c09096. 33475373

[ref26] Yamamoto S.; Back R. A. The Photolysis and Thermal Decomposition of Pyruvic Acid in the Gas Phase. Can. J. Chem. 1985, 63, 549–554. 10.1139/v85-089.

[ref27] Andreae M. O.; Talbot R. W.; Li S.-M. Atmospheric Measurements of Pyruvic and Formic Acid. J. Geophys. Res.: Atmos. 1987, 92, 6635–6641. 10.1029/JD092iD06p06635.

[ref28] Takahashi K.; Plath K. L.; Skodje R. T.; Vaida V. Dynamics of Vibrational Overtone Excited Pyruvic Acid in the Gas Phase: Line Broadening through Hydrogen-Atom Chattering. J. Phys. Chem. A 2008, 112, 7321–7331. 10.1021/jp803225c. 18637664

[ref29] Plath K. L.; Takahashi K.; Skodje R. T.; Vaida V. Fundamental and Overtone Vibrational Spectra of Gas-Phase Pyruvic Acid. J. Phys. Chem. A 2009, 113, 7294–7303. 10.1021/jp810687t. 19260671

[ref30] Harris A. E. R.; Doussin J.-F.; Carpenter B. K.; Vaida V. Gas-Phase Photolysis of Pyruvic Acid: The Effect of Pressure on Reaction Rates and Products. J. Phys. Chem. A 2016, 120, 10123–10133. 10.1021/acs.jpca.6b09058. 27992197

[ref31] Jacob D. J.; Wofsy S. C. Photochemistry of Biogenic Emissions over the Amazon Forest. J. Geophys. Res.: Atmos. 1988, 93, 1477–1486. 10.1029/JD093iD02p01477.

[ref32] Grosjean D.; Williams E. L.; Grosjean E. Atmospheric Chemistry of Isoprene and of its Carbonyl Products. Environ. Sci. Technol. 1993, 27, 830–840. 10.1021/es00042a004.

[ref33] Paulot F.; Crounse J. D.; Kjaergaard H. G.; Kroll J. H.; Seinfeld J. H.; Wennberg P. O. Isoprene Photooxidation: New Insights into the Production of Acids and Organic Nitrates. Atmos. Chem. Phys. 2009, 9, 1479–1501. 10.5194/acp-9-1479-2009.

[ref34] Raber W. H.; Moortgat G. K. Photooxidation of Selected Carbonyl Compounds in Air: Methyl Ethyl Ketone, Methyl Vinyl Ketone, Methacrolein and Methylglyoxal. In Progress and Problems in Atmospheric Chemistry; Barker J. R., Ed.; World Scientific Publishing: Singapore, 1995; pp 318–373.

[ref35] Jenkin M. E.; Cox R. A.; Emrich M.; Moortgat G. K. Mechanisms of the Cl-Atom-Initiated Oxidation of Acetone and Hydroxyacetone in Air. J. Chem. Soc., Faraday Trans. 1993, 89, 2983–2991. 10.1039/ft9938902983.

[ref36] Warneck P. Multi-Phase Chemistry of C_2_ and C_3_ Organic Compounds in the Marine Atmosphere. J. Atmos. Chem. 2005, 51, 119–159. 10.1007/s10874-005-5984-7.

[ref37] Grosjean D. Atmospheric Reactions of Ortho Cresol: Gas Phase and Aerosol Products. Atmos. Environ. 1984, 18, 1641–1652. 10.1016/0004-6981(84)90386-X.

[ref38] Praplan A. P.; Hegyi-Gaeggeler K.; Barmet P.; Pfaffenberger L.; Dommen J.; Baltensperger U. Online Measurements of Water-Soluble Organic Acids in the Gas and Aerosol Phase from the Photooxidation of 1,3,5-Trimethylbenzene. Atmos. Chem. Phys. 2014, 14, 8665–8677. 10.5194/acp-14-8665-2014.

[ref39] Horowitz A.; Meller R.; Moortgat G. K. The UV–vis Absorption Cross Sections of the α-Dicarbonyl Compounds: Pyruvic Acid, Biacetyl and Glyoxal. J. Photochem. Photobiol., A 2001, 146, 19–27. 10.1016/S1010-6030(01)00601-3.

[ref40] Carlton A. G.; Turpin B. J.; Lim H.-J.; Altieri K. E.; Seitzinger S. Link between Isoprene and Secondary Organic Aerosol (SOA): Pyruvic Acid Oxidation Yields Low Volatility Organic Acids in Clouds. Geophys. Res. Lett. 2006, 33, L06822 10.1029/2005GL025374.

[ref41] Tan Y.; Lim Y. B.; Altieri K. E.; Seitzinger S. P.; Turpin B. J. Mechanisms Leading to Oligomers and SOA through Aqueous Photooxidation: Insights from OH Radical Oxidation of Acetic Acid and Methylglyoxal. Atmos. Chem. Phys. 2012, 12, 801–813. 10.5194/acp-12-801-2012.

[ref42] Eugene A. J.; Guzman M. I. The Effects of Reactant Concentration and Air Flow Rate in the Consumption of Dissolved O_2_ during the Photochemistry of Aqueous Pyruvic Acid. Molecules 2019, 24, 1124 10.3390/molecules24061124. 30901878 PMC6470820

[ref43] Mekic M.; Liu J.; Zhou W.; Loisel G.; Cai J.; He T.; Jiang B.; Yu Z.; Lazarou Y. G.; Li X.; Brigante M.; Vione D.; Gligorovski S. Formation of Highly Oxygenated Multifunctional Compounds from Cross-Reactions of Carbonyl Compounds in the Atmospheric Aqueous Phase. Atmos. Environ. 2019, 219, 117046 10.1016/j.atmosenv.2019.117046.

[ref44] Grosjean D. Atmospheric Reactions of Pyruvic Acid. Atmos. Environ. 1983, 17, 2379–2382. 10.1016/0004-6981(83)90242-1.

[ref45] Winterhalter R.; Jensen N. R.; Magneron I.; Wirtz K.; Mellouki W.; Yuying M.; Tadic J.; Horowitz A.; Moortgat G. K.; Hjorth J. In Studies of the Photolysis of Pyruvic Acid: Products and Mechanism; Proceedings of the EUROTRAC-2 Symposium 2000 on “Transport and Chemical Transformation in the Troposphere”; Midgley P. M.; Reuther M.; Williams M., Eds.; Springer: Garmisch Partenkirchen, 2001.

[ref46] Mellouki A.; Mu Y. On the Atmospheric Degradation of Pyruvic Acid in the Gas Phase. J. Photochem. Photobiol., A 2003, 157, 295–300. 10.1016/S1010-6030(03)00070-4.

[ref47] Petersen-Sonn E. A.; Jespersen M. F.; Johnson M. S.; Mikkelsen K. V. Mechanistic Insights into UV Spectral Changes of Pyruvic Acid and Pyruvate Part 1: Interaction with Water Molecules. Int. J. Phys. Res. Appl. 2024, 7, 100–107. 10.29328/journal.ijpra.1001092.

[ref48] Zhang J.; Dolg M. ABCluster: The Artificial Bee Colony Algorithm for Cluster Global Optimization. Phys. Chem. Chem. Phys. 2015, 17, 24173–24181. 10.1039/C5CP04060D. 26327507

[ref49] Zhang J.; Dolg M. Global Optimization of Clusters of Rigid Molecules Using the Artificial Bee Colony Algorithm. Phys. Chem. Chem. Phys. 2016, 18, 3003–3010. 10.1039/C5CP06313B. 26738568

[ref50] Grimme S.; Bannwarth C.; Shushkov P. A Robust and Accurate Tight-Binding Quantum Chemical Method for Structures, Vibrational Frequencies, and Noncovalent Interactions of Large Molecular Systems Parametrized for All spd-Block Elements (*Z* = 1–86). J. Chem. Theory Comput. 2017, 13, 1989–2009. 10.1021/acs.jctc.7b00118. 28418654

[ref51] Bannwarth C.; Caldeweyher E.; Ehlert S.; Hansen A.; Pracht P.; Seibert J.; Spicher S.; Grimme S. Extended Tight-Binding Quantum Chemistry Methods. WIREs Comput. Mol. Sci. 2021, 11, e1493 10.1002/wcms.1493.

[ref52] Frisch M. Gaussian 16; revision B.01.; Gaussian Inc.: Wallingford CT., 2016.

[ref53] Neese F. Software Update: The ORCA Program System–Version 5.0. WIREs Comput. Mol. Sci. 2022, 12, e1606 10.1002/wcms.1606.

[ref54] Myllys N.; Elm J.; Halonen R.; Kurtén T.; Vehkamäki H. Coupled Cluster Evaluation of the Stability of Atmospheric Acid-Base Clusters with up to 10 Molecules. J. Phys. Chem. A 2016, 120, 621–630. 10.1021/acs.jpca.5b09762. 26771121

[ref55] Schmitz G.; Elm J. Assessment of the DLPNO binding energies of strongly non-covalent bonded atmospheric molecular clusters. ACS Omega 2020, 5, 7601–7612. 10.1021/acsomega.0c00436. 32280904 PMC7144154

[ref56] Jensen A. B.; Kubecka J.; Schmitz G.; Christiansen O.; Elm J. Massive Assessment of the Binding Energies of Atmospheric Molecular Clusters. J. Chem. Theory Comput. 2022, 18, 7373–7383. 10.1021/acs.jctc.2c00825. 36417753

[ref57] Jensen A. B.; Elm J. Massive Assessment of the Geometries of Atmospheric Molecular Clusters. J. Chem. Theory Comput. 2024, 20, 8549–8558. 10.1021/acs.jctc.4c01046. 39331672

[ref58] Kubečka J.; Besel V.; Neefjes I.; Knattrup Y.; Kurtén T.; Vehkamäki H.; Elm J. Computational Tools for Handling Molecular Clusters: Configurational Sampling, Storage, Analysis, and Machine Learning. ACS Omega 2023, 8, 45115–45128. 10.1021/acsomega.3c07412. 38046354 PMC10688175

[ref59] Elm J. An Atmospheric Cluster Database Consisting of Sulfuric Acid, Bases, Organics, and Water. ACS Omega 2019, 4, 10965–10974. 10.1021/acsomega.9b00860.

[ref60] Blair S. L.; Harris A. E. R.; Frandsen B. N.; Kjaergaard H. G.; Pangui E.; Cazaunau M.; Doussin J.-F.; Vaida V. Conformer-Specific Photolysis of Pyruvic Acid and the Effect of Water. J. Phys. Chem. A 2020, 124, 1240–1252. 10.1021/acs.jpca.9b10613. 31976674

[ref61] Borba A.; Gómez-Zavaglia A.; Lapinski L.; Fausto R. Rotational Isomers of Lactic Acid: First Experimental Observation of Higher Energy Forms. Phys. Chem. Chem. Phys. 2004, 6, 2101–2108. 10.1039/B316642B.

[ref62] Zhang J. Atom Typing Using Graph Representation Learning: How Do Models Learn Chemistry. J. Chem. Phys. 2022, 156, 204108 10.1063/5.0095008. 35649855

[ref63] Hirshfeld F. L. Bonded-Atom Fragments for Describing Molecular Charge Densities. Theor. Chim. Acta 1977, 44, 129–138. 10.1007/BF00549096.

[ref64] Marenich A. V.; Jerome S. V.; Cramer C. J.; Truhlar D. G. Charge Model 5: An Extension of Hirshfeld Population Analysis for the Accurate Description of Molecular Interactions in Gaseous and Condensed Phases. J. Chem. Theory Comput. 2012, 8, 527–541. 10.1021/ct200866d. 26596602

[ref65] Kubečka J.; Besel V.; Kurtén T.; Myllys N.; Vehkamaki H. Configurational Sampling of Noncovalent (Atmospheric) Molecular Clusters: Sulfuric Acid and Guanidine. J. Phys. Chem. A 2019, 123, 6022–6033. 10.1021/acs.jpca.9b03853. 31273989

[ref66] Grimme S. Supramolecular Binding Thermodynamics by Dispersion-Corrected Density Functional Theory. Chem. - Eur. J. 2012, 18, 9955–9964. 10.1002/chem.201200497. 22782805

[ref67] Luchini G.; Alegre-Requena J. V.; Funes-Ardoiz I.; Paton R. S. GoodVibes: Automated Thermochemistry for Heterogeneous Computational Chemistry Data. F1000Research 2020, 9, 291 10.12688/f1000research.22758.1.

[ref68] Dunn M. E.; Evans T. M.; Kirschner K. N.; Shields G. C. Prediction of Accurate Anharmonic Experimental Vibrational Frequencies for Water Clusters, (H2O)n, n = 2–5. J. Phys. Chem. A 2006, 110, 303–309. 10.1021/jp054958y. 16392869 PMC2548414

[ref69] Temelso B.; Archer K. A.; Shields G. C. Benchmark Structures and Binding Energies of Small Water Clusters with Anharmonicity Corrections. J. Phys. Chem. A 2011, 115, 12034–12046. 10.1021/jp2069489. 21910428

[ref70] Temelso B.; Shields G. C. The Role of Anharmonicity in Hydrogen-Bonded Systems: The Case of Water Cluster. J. Chem. Theory Comput. 2011, 7, 2804–2817. 10.1021/ct2003308. 26605472

[ref71] Riplinger C.; Neese F. An Efficient and Near Linear Scaling Pair Natural Orbital Based Local Coupled Cluster Method. J. Chem. Phys. 2013, 138, 034106 10.1063/1.4773581. 23343267

[ref72] Riplinger C.; Sandhoefer B.; Hansen A.; Neese F. Natural Triple Excitations in Local Coupled Cluster Calculations with Pair Natural Orbitals. J. Chem. Phys. 2013, 139, 134101 10.1063/1.4821834. 24116546

[ref73] Elm J.; Kubečka J.; Besel V.; Jääskeläinen M. J.; Halonen R.; Kurtén T.; Vehkamäki H. Modeling the Formation and Growth of Atmospheric Molecular Clusters: A Review. J. Aerosol Sci. 2020, 149, 105621 10.1016/j.jaerosci.2020.105621.

[ref74] Dennington A.; Keith T. A.; Millam J. M. GaussView; Version 6; Semichem Inc.: Shawnee Mission KS, 2019.

[ref75] Dzidic I.; Kebarle P. Hydration of the Alkali Ions in the Gas Phase. Enthalpies and Entropies of Reactions M^+^(H_2_O)_*n*−1_ + H_2_O = M^+^(H_2_O)_*n*_. J. Phys. Chem. A 1970, 74, 1466–1474. 10.1021/j100702a013.

[ref76] Schulz C. P.; Haugstätter R.; Tittes H. U.; Hertel I. V. Free Sodium-Water Clusters. Phys. Rev. Lett. 1986, 57, 1703 10.1103/PhysRevLett.57.1703. 10033523

[ref77] Schulz C. P.; Haugstätter R.; Tittes H. U.; Hertel I. V. Free Sodium-Water Clusters: Photoionisation Studies in a Pulsed Molecular Beam Source. Z. Phys. D:At., Mol. Clusters 1988, 10, 279–290. 10.1007/BF01384862.

[ref78] Hertel I. V.; Hüglin C.; Nitsch C.; Schulz C. P. Photoionization of Na(NH_3_)_*n*_ and Na(H_2_O)_*n*_ Clusters: A Step Towards the Liquid Phase?. Phys. Rev. Lett. 1991, 67, 1767 10.1103/PhysRevLett.67.1767. 10044242

[ref79] Patwari G. N.; Lisy J. M. Mimicking the Solvation of Aqueous Na^+^ in the Gas Phase. J. Chem. Phys. 2003, 118, 8555–8558. 10.1063/1.1574018.

[ref80] Vaden T. D.; Weinheimer C. J.; Lisy J. M. Evaporatively Cooled M^+^(H_2_O)Ar Cluster Ions: Infrared Spectroscopy and Internal Energy Simulations. J. Chem. Phys. 2004, 121, 3102–3107. 10.1063/1.1774157. 15291620

[ref81] Mancinelli R.; Botti A.; Bruni F.; Ricci M. A.; Soper A. K. Hydration of Sodium, Potassium, and Chloride Ions in Solution and the Concept of Structure Maker/Breaker. J. Phys. Chem. B 2007, 111, 13570–13577. 10.1021/jp075913v. 17988114

[ref82] Perez P.; Lee W. K.; Prohofsky E. W. Study of Hydration of the Na^+^ Ion Using a Polarizable Water Model. J. Chem. Phys. 1983, 79, 388–392. 10.1063/1.445534.

[ref83] Arbman M.; Siegbahn H.; Pettersson L.; Siegbahn P. Core Electron Binding Energies and Auger Electron Energies of Solvated Clusters: A Computational Study. Mol. Phys. 1985, 54, 1149–1160. 10.1080/00268978500100911.

[ref84] Lybrand T. P.; Kollman P. A. Water–Water and Water–Ion Potential Functions Including Terms for Many Body Effects. J. Chem. Phys. 1985, 83, 2923–2933. 10.1063/1.449246.

[ref85] Cieplak P.; Lybrand T. P.; Kollman P. A. Calculation of Free Energy Changes in Ion-Water Clusters Using Nonadditive Potentials and the Monte Carlo Method. J. Chem. Phys. 1987, 86, 6393–6403. 10.1063/1.452428.

[ref86] Probst M. M. A Study of the Additivity of Interactions in Cation-Water Systems. Chem. Phys. Lett. 1987, 137, 229–232. 10.1016/0009-2614(87)80210-5.

[ref87] Bauschlicher C. W. Jr.; Langhoff S. R.; Partridge H.; Rice J. E.; Komornicki A. A Theoretical Study of Na(H_2_O)_*n*_^+^ (*n* = 1 – 4). J. Chem. Phys. 1991, 95, 5142–5148. 10.1063/1.461682.

[ref88] Dang L. X.; Rice J. E.; Caldwell J.; Kollman P. A. Ion Solvation in Polarizable Water: Molecular Dynamics Simulations. J. Am. Chem. Soc. 1991, 113, 2481–2486. 10.1021/ja00007a021.

[ref89] Perera L.; Berkowitz M. L. Many-Body Effects in Molecular Dynamics Simulations of Na^+^(H_2_O)_*n*_ and Cl^–^(H_2_O)_*n*_ Clusters. J. Chem. Phys. 1991, 95, 1954–1963. 10.1063/1.460992.

[ref90] Hashimoto K.; Morokuma K. Ab Initio Molecular Orbital Study of Na(H_2_O)_*n*_ (n = 1–6) Clusters and Their Ions. Comparison of Electronic Structure of the “Surface” and “Interior” Complexes. J. Am. Chem. Soc. 1994, 116, 11436–11443. 10.1021/ja00104a024.

[ref91] Glendening E. D.; Feller D. Cation-Water Interactions: The M^+^(H_2_O)_*n*_ Clusters for Alkali Metals, M = Li, Na, K, Rb, and Cs. J. Phys. Chem. A 1995, 99, 3060–3067. 10.1021/j100010a015.

[ref92] Kim J.; Lee S.; Cho S. J.; Mhin B. J.; Kim K. S. Structures, Energetics, and Spectra of Aqua-Sodium (I): Thermodynamic Effects and Nonadditive Interactions. J. Chem. Phys. 1995, 102, 839–849. 10.1063/1.469199.

[ref93] Ramaniah L. M.; Bernasconi M.; Parrinello M. Density-Functional Study of Hydration of Sodium in Water Clusters. J. Chem. Phys. 1998, 109, 6839–6843. 10.1063/1.477250.

[ref94] Carrillo-Tripp M.; Saint-Martin H.; Ortega-Blake I. A Comparative Study of the Hydration of Na^+^ and K^+^ with Refined Polarizable Model Potentials. J. Chem. Phys. 2003, 118, 7062–7073. 10.1063/1.1559673.

[ref95] Lee H. M.; Tarakeshwar P.; Park J.; Kołaski M. R.; Yoon Y. J.; Yi H.-B.; Kim W. Y.; Kim K. S. Insights into the Structures, Energetics, and Vibrations of Monovalent Cation-(Water)_1–6_ Clusters. J. Phys. Chem. A 2004, 108, 2949–2958. 10.1021/jp0369241.

[ref96] Rao J. S.; Dinadayalane T. C.; Leszczynski J.; Sastry G. N. Comprehensive Study on the Solvation of Mono- and Divalent Metal Cations: Li^+^, Na^+^, K^+^, Be^2+^, Mg^2+^ and Ca^2+^. J. Phys. Chem. A 2008, 112, 12944–12953. 10.1021/jp8032325. 18834092

[ref97] Neela Y. I.; Mahadevi A. S.; Sastry G. N. First Principles Study and Database Analyses of Structural Preferences for Sodium Ion (Na^+^) Solvation and Coordination. Struct. Chem. 2013, 24, 67–79. 10.1007/s11224-012-0032-0.

[ref98] Biring S. K.; Sharma R.; Misra R.; Chaudhury P. Structural and Infra Red Spectroscopic Aspects of Ion-Water Clusters: A Study Based on a Combined Stochastic and Quantum Chemical Approach. J. Cluster Sci. 2013, 24, 715–737. 10.1007/s10876-013-0565-4.

[ref99] Dinh P. M.; Gao C. Z.; Klüpfel P.; Reinhard P.-G.; Suraud E.; Vincendon M.; Wang J.; Zhang F. S. A Density Functional Theory Study of Na(H_2_O)_*n*_: An Example of the Impact of Self-Interaction Corrections. Eur. Phys. J. D 2014, 68, 239 10.1140/epjd/e2014-40816-1.

[ref100] Soniat M.; Rogers D. M.; Rempe S. B. Dispersion- and Exchange-Corrected Density Functional Theory for Sodium Ion Hydration. J. Chem. Theory Comput. 2015, 11, 2958–2967. 10.1021/acs.jctc.5b00357. 26575733

[ref101] Fifen J. J.; Agmon N. Structure and Spectroscopy of Hydrated Sodium Ions at Different Temperatures and the Cluster Stability Rules. J. Chem. Theory Comput. 2016, 12, 1656–1673. 10.1021/acs.jctc.6b00038. 26913993

[ref102] Wang P.; Shi R.; Su Y.; Tang L.; Huang X.; Zhao J. Hydrated Sodium Ion Clusters [Na^+^(H_2_O)_*n*_ (n = 1–6)]: An Ab Initio Study on Structures and Non-Covalent Interaction. Front. Chem. 2019, 7, 624 10.3389/fchem.2019.00624. 31572714 PMC6751288

[ref103] Keesee R. G.; Castleman A. W. Jr Gas-Phase Studies of Hydration Complexes of Cl^–^ and I^–^ and Comparison to Electrostatic Calculations in the Gas Phase. Chem. Phys. Lett. 1980, 74, 139–142. 10.1016/0009-2614(80)85031-7.

[ref104] Likholyot A.; Hovey J. K.; Seward T. M. Experimental and Theoretical Study of Hydration of Halide Ions. Geochim. Cosmochim. Acta 2005, 69, 2949–2958. 10.1016/j.gca.2004.12.014.

[ref105] Xantheas S. S. Quantitative Description of Hydrogen Bonding in Chloride-Water Clusters. J. Phys. Chem. A 1996, 100, 9703–9713. 10.1021/jp960779s.

[ref106] Masamura M. Structures, Energetics, and Spectra of Cl^–^(H_2_O)_*n*_ Clusters, n = 1–6: Ab Initio Study. J. Phys. Chem. A 2002, 106, 8925–8932. 10.1021/jp014700h.

[ref107] Xantheas S. S.; Dunning T. H. Jr Ab Initio Studies of Cyclic Water Clusters (H_2_O)_*n*_, n = 1–6. I. Optimal Structures and Vibrational Spectra. J. Chem. Phys. 1993, 99, 8774–8792. 10.1063/1.465599.

[ref108] Xantheas S. S. Ab Initio Studies of Cyclic Water Clusters (H_2_O)_*n*_, n = 1–6. II. Analysis of Many-Body Interactions. J. Chem. Phys. 1994, 100, 7523–7534. 10.1063/1.466846.

[ref109] Xantheas S. S. Ab Initio Studies of Cyclic Water Clusters (H_2_O)_*n*_, n = 1–6. III. Comparison of Density Functional with MP2 Results. J. Chem. Phys. 1995, 102, 4505–4517. 10.1063/1.469499.

[ref110] Shemesh D.; Luo M.; Grassian V. H.; Gerber R. B. Absorption Spectra of Pyruvic Acid in Water: Insights from Calculations for Small Hydrates and Comparison to Experiment. Phys. Chem. Chem. Phys. 2020, 22, 12658–12670. 10.1039/D0CP01810D. 32458893

[ref111] Kildgaard J. V.; Mikkelsen K. V.; Bilde M.; Elm J. Hydration of Atmospheric Molecular Clusters II: Organic Acid-Water Clusters. J. Phys. Chem. A 2018, 122, 8549–8556. 10.1021/acs.jpca.8b07713. 30351100

[ref112] Li S.; Kjaergaard H. G.; Du L. Infrared Spectroscopic Probing of Dimethylamine Clusters in an Ar Matrix. J. Environ. Sci. 2016, 40, 51–59. 10.1016/j.jes.2015.09.012. 26969545

[ref113] Du L.; Mackeprang K.; Kjaergaard H. G. Fundamental and Overtone Vibrational Spectroscopy, Enthalpy of Hydrogen Bond Formation and Equilibrium Constant Determination of the Methanol-Dimethylamine Complex. Phys. Chem. Chem. Phys. 2013, 15, 10194–10206. 10.1039/C3CP50243K. 23695525

