# Supplementary material for: Modeling Local Aerosol Surface Environments: Clustering of Pyruvic Acid Analogs, Water, and Na+, Cl– Ions
Source: ACS Omega. 2025 Jan 2;10(1):1470–85. doi: 10.1021/acsomega.4c09196 (PMC11740629; doi:10.1021/acsomega.4c09196)
Supplement: Supplementary file 1 — ao4c09196_si_001.pdf [file ao4c09196_si_001.pdf]

Supporting Information: Modeling Local Aerosol Surface Environments: Clustering of Pyruvic Acid Analogs, Water, and Na^{+}, Cl^{-} Ions

Georg Baadsgaard Trolle, Jakub kubečka, and Jonas Elm*

Department of Chemistry, Aarhus University, Langelandsgade 140, 8000 Aarhus C, Denmark

E-mail: jelm@chem.au.dk

Phone: +45 28938085

S1 Computational Details

S1.1 Assessment of Pyruvic Acid Conformers

Prior to configurational sampling, we carried out a detailed benchmarking study. The motivation for performing benchmarking was due to the water, which we postulated would have a significant influence on the binding free energies of the clusters, as the water molecules can engage in many different hydrogen bonding networks with the central organic acid monomer—especially at higher water counts. Furthermore, benchmarking facilitates proper treatment of the key interactions, which operate internally in the different conformers of pyruvic acid, namely the intramolecular hydrogen bonding between the α-carbonyl group and the carboxylic O–H proton of the **Tc** conformer (see Figure 1**B**). The sets of benchmarking studies, which were conducted in this study are six-fold: i) Pyruvic acid alone; ii) pyruvic acid and one water; iii) pyruvic acid and Na^+; iv) pyruvic acid and Cl^-; v) Na^+ and one water, and vi) Cl^- and one water. The methods that were probed as a part of the benchmarking study include semi-empirical methods, DFT methods, and high-level quantum chemically correlated methods and are provided in Table S1.

Table S1: Methods probed as a part of the benchmarking study.

Semi-empirical methods	DFT functionals	Correlated methods
GFN1-xTB	ωB97X-D	CCSD
GFN2-xTB	M06-2X	DLPNO-CCSD(T_0)*
B97-3c	PW91	MP2**
PBEh-3c		
r^2SCAN-3c		

* The F12 explicit correlation was also included for this method.
** Benchmarking with this method was only performed for the pyruvic acid monomer.

For the DFT functionals and the correlated methods in Table S1 in the benchmarking of the pyruvic acid monomer alone, we employed the two Pople-style basis sets

6-31++G(d,p) and 6-311++G(3df,3pd) as well as the Dunning-type basis sets aug-cc-pVDZ, aug-cc-pVTZ, and aug-cc-pVQZ. For this benchmarking, we consistently performed geometry optimizations and vibrational frequency calculations for each method and level of theory except for high-level CCSD(T_0) correlated methods where we computed single-point electronic energies on the MP2/aug-cc-pVQZ geometries. Furthermore, we employed ArbAlign to calculate root mean squared deviations (RMSD) of all four pyruvic acid equilibrium conformations between all DFT methods and the geometries obtained at MP2/aug-cc-pVQZ level of theory. The methods were subsequently used in the configurational sampling protocol for the gas-phase clusters.

S1.2 Assessment of Pyruvic Acid Interaction Energies

For the benchmarkings of the pyruvic acid monomer with ion and water with ion, we employed only the 6-31++G(d,p) and aug-cc-pVTZ basis sets and only computed single-point electronic energies on the ωB97X-D/aug-cc-pVTZ geometries of the four pyruvic acid conformers. The benchmarked methods at the semi-empirical, DFT, and correlated quantum chemical level of theory were then selected based on the most accurate calculations of the expected binding free energies and interaction single-point energies of the four conformers of pyruvic acid and based on previous literature.

S2 Benchmarking results

For the benchmarking, the energies for the **Tc** conformer of PA were normalized to zero across all methods in order to compare the relative conformational energies of the PA conformers inherent to a specific method with literature values. The relative thermal free corrections and relative single-point electronic energies with respect to the **Tc** conformer are provided here.

To emphasize that the DLPNO-CCSD(T_0)/aug-cc-pVTZ//ωB97X-D/6-31++G(d,p) is

an adequate level of theory for the systems at hand, we carried out a benchmark study of the PA monomer. Table S2 below presents the calculated relative energies of the PA conformers at semi-empirical, DFT, and correlated wavefunction levels of theory, respectively.

We found that GFN1-xTB was the optimal semi-empirical method to employ for the subsequent CS as we observed this method to adequately reproduce the **Cc** conformer as the highest energy conformer and conversely the **Tc** conformer as the lowest energy conformer in conformity to previous literature. Furthermore, the two conformers were observed to be separated by a significant relative free energy with the **Ct** and **Tt** conformers adopting a relative free energy in between these two extremes thereby supporting our choice of method. For the benchmarked DFT methodologies, the ωB97X-D/6-31++G(d,p) methodology was found to reproduce the relative conformational energies most accurately in addition to its low computational cost. The benchmarking of the final high-level correlated methods, DLPNO-CCSD(T_0)/aug-cc-pVTZ with the RI-JK approximation, was found to be the most accurate even though it was not the most computationally efficient method of the benchmarked, we considered accuracy to be superior to cost at this level of theory.

Additionally, the DFT functionals, ωB97X-D and M06-2X, are shown to perform similarly and both functionals provide equilibrium geometries, which deviates only slightly from the MP2/aug-cc-pVQZ reference on the basis of the RMSD comparisons as is shown in Figure S1. Furthermore, ωB97X-D and M06-2X are both observed to outperform PW91, which performs poorly in combination with all the probed basis sets. Therefore, we consider ωB97X-D/6-31++G(d,p) to be an adequate methodology as it is both accurate and computationally inexpensive.

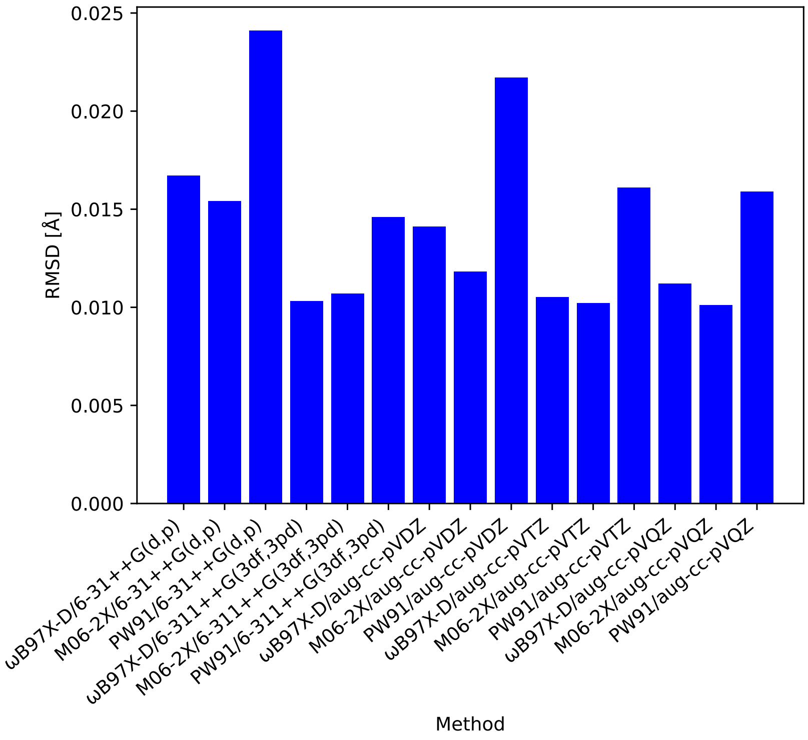

Figure S1: Averaged root mean square displacements (RMSD) over the four PA conformer geometries obtained with the different DFT methodologies against the RMSD of the MP2/aug-cc-pVQZ reference geometries.

To further corroborate the conclusions drawn from the benchmark study of the PA monomer, we conducted benchmark studies on the PA monomer with one water (Table S3), the PA monomer with Na^+ (Table S4), the PA monomer with Cl^- (Table S5), water with Na^+ (Table S6) as well as water with Cl^- (Table S7) in order to probe the interaction energies between the components of the studied systems. The above referenced tables present the calculated binding energies for these systems at different levels of theory. All binding energies were calculated on top of the ωB97X-D/6-31++G(d,p) geometry of the PA conformers. From the benchmark study performed for the PA monomer with one water molecule, the **Ct** conformer was observed to produce the lowest binding energies while for the benchmark studies performed for the PA monomer with Na^+ or Cl^- on the contrary, the **Cc** conformer was observed to produce the lowest

binding energies. The benchmark studies performed for the water with Na^{+} or Cl^{-} reveal that both the PA monomer and the ions contribute equally much to the binding energies.

Table S2: Relative free energies obtained at semi-empirical, DFT, CCSD, and MP2 level of theory and relative single-point energies obtained at CCSD(T_0) level of theory in kcal/mol with respect to the lowest energy PA conformer **Tc**. Note that the CCSD(T_0) single-point energies were computed on the MP2/aug-cc-pVQZ geometry of the conformers.

Semi-empirical methods				
	**Tc**	**Tt**	**Ct**	**Cc**
GFN1-xTB	0	0.006	2.406	11.958
GFN2-xTB	0	1.212	2.597	10.343
B97-3c	0	2.798	4.947	11.009
PBEh-3c	0	1.278	3.156	10.566
r^2SCAN-3c	0	2.719	4.989	10.666
DFT methodologies				
	**Tc**	**Tt**	**Ct**	**Cc**
ωB97X-D/aug-cc-pVDZ	0	2.214	3.702	11.220
ωB97X-D/aug-cc-pVTZ	0	2.321	3.806	9.029
ωB97X-D/aug-cc-pVQZ	0	2.250	3.632	10.990
ωB97X-D/6-31++G(d,p)	0	1.758	3.019	11.624
ωB97X-D/6-311++G(3df,3pd)	0	2.201	3.500	11.062
M06-2X/aug-cc-pVDZ	0	2.112	3.668	9.782
M06-2X/aug-cc-pVTZ	0	2.187	3.771	9.937
M06-2X/aug-cc-pVQZ	0	2.177	3.774	9.859
M06-2X/6-31++G(d,p)	0	1.530	2.845	11.384
M06-2X/6-311++G(3df,3pd)	0	2.113	3.666	9.610
PW91/aug-cc-pVDZ	0	2.673	4.015	11.106
PW91/aug-cc-pVTZ	0	2.658	3.945	9.195
PW91/aug-cc-pVQZ	0	2.615	3.769	10.949
PW91/6-31++G(d,p)	0	2.222	3.414	11.404
PW91/6-311++G(3df,3pd)	0	2.620	3.799	11.027
Correlated methods				
	**Tc**	**Tt**	**Ct**	**Cc**
CCSD/aug-cc-pVDZ	0	1.694	2.907	10.752
CCSD/cc-pVTZ†	0	2.414	4.182	10.944
CCSD/6-31++G(d,p)	0	0.144	3.419	11.257
DLPNO-CCSD(T_0)/aug-cc-pVDZ	0	2.473	3.977	10.414
DLPNO-CCSD(T_0)-F12/aug-cc-pVDZ	0	2.879	4.394	10.563
RI-CCSD(T_0)-F12/aug-cc-pVDZ	0	2.705	4.340	10.666
DLPNO-CCSD(T_0)/aug-cc-pVTZ	0	2.592	4.146	10.454
DLPNO-CCSD(T_0)-F12/aug-cc-pVTZ	0	2.755	4.393	10.727
RI-CCSD(T_0)-F12/aug-cc-pVTZ	0	2.775	4.415	10.759
DLPNO-CCSD(T_0)/aug-cc-pVQZ	0	2.686	4.257	10.600
DLPNO-CCSD(T_0)-F12/aug-cc-pVQZ	0	2.782	4.437	10.737
RI-CCSD(T_0)-F12/aug-cc-pVQZ	0	2.768	4.403	10.767
MP2/aug-cc-pVDZ	0	1.955	3.160	10.814
MP2/aug-cc-pVTZ	0	2.152	3.392	9.686
MP2/aug-cc-pVQZ	0	2.189	3.427	9.669

† The calculations were continued in ORCA on top of the non-converged aug-cc-pVTZ geometries obtained for the PA conformers in Gaussian 16.

Table S3: Binding free energies obtained at DFT and CCSD level of theory and interaction single-point energies obtained at semi-empirical and CCSD(T_0) level of theory in kcal/mol for the PA monomer with one water molecule.

Semi-empirical methods			
	**Tc**	**Tt**	**Ct**
GFN1-xTB	−8.495	−3.857	−11.029
GFN2-xTB	−7.602	−3.564	−10.201
B97-3c	−8.027	−4.626	−11.785
PBEh-3c	−8.893	−3.529	−12.017
r^2SCAN-3c	−7.948	−4.757	−11.413
DFT methodologies			
	**Tc**	**Tt**	**Ct**
ωB97X-D/aug-cc-pVTZ	3.390	6.416	1.153
ωB97X-D/6-31++G(d,p)	0.745	3.258	−1.620
M06-2X/aug-cc-pVTZ	2.211	5.575	−0.186
M06-2X/6-31++G(d,p)	−1.087	3.116	−2.688
PW91/aug-cc-pVTZ	2.715	7.056	0.519
PW91/6-31++G(d,p)	2.165	6.512	−0.717
Correlated methods			
	**Tc**	**Tt**	**Ct**
CCSD/aug-cc-pVDZ	3.663	7.202	2.322
CCSD/6-31++G(d,p)	3.863	7.031	−0.136
DLPNO-CCSD(T_0)/aug-cc-pVDZ(loose PNO)	−8.090	−4.906	−10.660
DLPNO-CCSD(T_0)/aug-cc-pVDZ(normal PNO)	−8.314	−5.230	−11.243
DLPNO-CCSD(T_0)/aug-cc-pVDZ(tight PNO)	−8.536	−5.411	−11.519
DLPNO-CCSD(T_0)/aug-cc-pVTZ(loose PNO)	−7.666	−4.540	−10.592
DLPNO-CCSD(T_0)/aug-cc-pVTZ(normal PNO)	−7.980	−4.872	−11.217
DLPNO-CCSD(T_0)/aug-cc-pVQZ(loose PNO)	−7.413	−4.409	−10.674
DLPNO-CCSD(T_0)-F12/cc-pVDZ-F12 (loose PNO)	−6.917	−3.985	−9.944
DLPNO-CCSD(T_0)-F12/cc-pVDZ-F12 (normal PNO)	−7.195	−4.372	−10.595
DLPNO-CCSD(T_0)-F12/cc-pVDZ-F12 (tight PNO)	−7.715	−4.685	−11.246
DLPNO-CCSD(T_0)-F12/cc-pVTZ-F12 (loose PNO)	−7.320	−4.285	−10.556
DLPNO-CCSD(T_0)-F12/cc-pVTZ-F12 (normal PNO)	−7.542	−4.472	−11.167

Note that the calculations for the **Cc** conformer of PA were not performed as the initial geometry optimization at DFT level did not converge for this. CCSD/aug-cc-pVTZ, DLPNO-CCSD(T_0)/aug-cc-pVTZ with tight PNO, DLPNO-CCSD(T_0)/aug-cc-pVQZ with normal PNO, DLPNO-CCSD(T_0)/aug-cc-pVQZ with tight PNO, and DLPNO-CCSD(T_0)-F12/cc-pVTZ-F12 with tight PNO did not converge for all of the PA conformers and are thus not included here.

Table S4: Binding free energies obtained at semi-empirical, DFT, and CCSD level of theory and interaction single-point energies obtained at CCSD(T_0) level of theory in kcal/mol for the PA monomer with one sodium ion.

Semi-empirical methods				
	**Tc**	**Tt**	**Ct**	**Cc**
GFN1-xTB	−31.119	−32.647	−41.161	−41.139
GFN2-xTB	−39.124	−39.804	−48.608	−48.443
B97-3c	−28.392	−31.815	−39.810	−39.830
PBEh-3c	−32.274	−35.906	−44.736	−44.725
r^2SCAN-3c	−28.736	−32.378	−41.067	−41.079
DFT methodologies				
	**Tc**	**Tt**	**Ct**	**Cc**
ωB97X-D/aug-cc-pVTZ	−20.330	−21.904	−29.767	−29.172
ωB97X-D/6-31++G(d,p)	−21.900	−23.558	−30.787	−32.249
M06-2X/aug-cc-pVTZ	−21.412	−23.981	−32.003	−32.928
M06-2X/6-31++G(d,p)	−22.586	−25.348	−32.665	−33.731
PW91/aug-cc-pVTZ	−21.018	−22.614	−30.276	−29.965
PW91/6-31++G(d,p)	−19.793	−22.767	−29.862	−31.372
Correlated methods				
	**Tc**	**Tt**	**Ct**	**Cc**
CCSD/aug-cc-pVDZ	−17.138	−19.727	−23.989	−27.548
CCSD/6-31++G(d,p)	−20.176	−20.411	−28.224	−28.577
DLPNO-CCSD(T_0)/aug-cc-pVDZ(loose PNO)	−30.310	−32.665	−39.468	−39.742
DLPNO-CCSD(T_0)/aug-cc-pVDZ(normal PNO)	−30.597	−33.081	−39.861	−40.031
DLPNO-CCSD(T_0)/aug-cc-pVDZ(tight PNO)	−30.696	−33.086	−39.844	−40.012
DLPNO-CCSD(T_0)/aug-cc-pVTZ(loose PNO)	−31.071	−34.679	−41.967	−42.054
DLPNO-CCSD(T_0)/aug-cc-pVTZ(normal PNO)	−31.301	−35.222	−42.174	−42.216
DLPNO-CCSD(T_0)/aug-cc-pVQZ(loose PNO)	−49.119	−61.911	−67.702	−67.515
DLPNO-CCSD(T_0)-F12/cc-pVDZ-F12 (loose PNO)	−27.575	−29.849	−37.616	−37.683
DLPNO-CCSD(T_0)-F12/cc-pVDZ-F12 (normal PNO)	−27.530	−29.978	−38.111	−38.171
DLPNO-CCSD(T_0)-F12/cc-pVDZ-F12 (tight PNO)	−27.436	−30.386	−38.469	−38.366
DLPNO-CCSD(T_0)-F12/cc-pVTZ-F12 (loose PNO)	−28.196	−30.793	−39.295	−38.931

CCSD/aug-cc-pVTZ, DLPNO-CCSD(T_0)/aug-cc-pVTZ with tight PNO, DLPNO-CCSD(T_0)/aug-cc-pVQZ with normal PNO, DLPNO-CCSD(T_0)/aug-cc-pVQZ with tight PNO, DLPNO-CCSD(T_0)-F12/cc-pVTZ-F12 with normal PNO, and DLPNO-CCSD(T_0)-F12/cc-pVTZ-F12 with tight PNO did not converge for all of the PA conformers and are thus not included here.

Table S5: Binding free energies obtained at semi-empirical, DFT, and CCSD level of theory and interaction single-point energies obtained at CCSD(T_0) level of theory in kcal/mol for the PA monomer with one chloride ion.

Semi-empirical methods				
	**Tc**	**Tt**	**Ct**	**Cc**
GFN1-xTB	−23.127	−20.948	−22.697	−34.408
GFN2-xTB	−27.109	−23.651	−24.752	−36.538
B97-3c	−32.575	−30.411	−31.782	−43.476
PBEh-3c	−39.569	−35.361	−37.775	−49.992
r^2SCAN-3c	−30.287	−28.433	−30.227	−41.301
DFT methodologies				
	**Tc**	**Tt**	**Ct**	**Cc**
ωB97X-D/aug-cc-pVTZ	−19.240	−17.734	−19.225	−28.262
ωB97X-D/6-31++G(d,p)	−20.001	−18.897	−20.482	−31.613
M06-2X/aug-cc-pVTZ	−20.376	−18.718	−20.653	−30.306
M06-2X/6-31++G(d,p)	−20.453	−18.794	−20.821	−31.821
PW91/aug-cc-pVTZ	1556.168	1555.967	1555.093	1546.971
PW91/6-31++G(d,p)	−19.552	−18.658	−20.068	−30.956
Correlated methods				
	**Tc**	**Tt**	**Ct**	**Cc**
CCSD/6-31++G(d,p)	−19.321	−15.896	−19.934	−30.575
DLPNO-CCSD(T_0)/aug-cc-pVDZ(loose PNO)	−26.296	−23.739	−25.756	−36.372
DLPNO-CCSD(T_0)/aug-cc-pVDZ(normal PNO)	−26.723	−23.927	−25.941	−37.151
DLPNO-CCSD(T_0)/aug-cc-pVDZ(tight PNO)	−26.654	−24.122	−26.147	−37.064
DLPNO-CCSD(T_0)/aug-cc-pVTZ(loose PNO)	−26.800	−24.428	−26.696	−37.176
DLPNO-CCSD(T_0)/aug-cc-pVQZ(loose PNO)	−26.474	−24.255	−26.408	−37.017
DLPNO-CCSD(T_0)-F12/cc-pVDZ-F12 (loose PNO)	−27.194	−25.017	−27.079	−37.896
DLPNO-CCSD(T_0)-F12/cc-pVDZ-F12 (normal PNO)	−27.386	−25.017	−27.079	−37.896
DLPNO-CCSD(T_0)-F12/cc-pVDZ-F12 (tight PNO)	−27.340	−25.639	−27.496	−37.843
DLPNO-CCSD(T_0)-F12/cc-pVTZ-F12 (loose PNO)	−26.790	−24.630	−26.852	−37.263
DLPNO-CCSD(T_0)-F12/cc-pVTZ-F12 (normal PNO)	−27.516	−25.190	−27.422	−38.268

CCSD/aug-cc-pVDZ, CCSD/aug-cc-pVTZ, DLPNO-CCSD(T_0)/aug-cc-pVTZ with normal PNO, DLPNO-CCSD(T_0)/aug-cc-pVTZ with tight PNO, DLPNO-CCSD(T_0)/aug-cc-pVQZ with normal PNO, DLPNO-CCSD(T_0)/aug-cc-pVQZ with tight PNO, and DLPNO-CCSD(T_0)-F12/cc-pVTZ-F12 with tight PNO did not converge for all of the PA conformers and are thus not included here.

Table S6: Binding free energies obtained at semi-empirical, DFT, and CCSD level of theory and interaction single-point energies obtained at CCSD(T_0) level of theory in kcal/mol for one water molecule with one sodium ion.

Semi-empirical methods	
	**System**
GFN1-xTB	−23.202
GFN2-xTB	−30.161
B97-3c	−24.640
PBEh-3c	−31.462
r^2SCAN-3c	−24.776
DFT methodologies	
	**System**
ωB97X-D/aug-cc-pVTZ	−16.222
ωB97X-D/6-31++G(d,p)	−18.228
M06-2X/aug-cc-pVTZ	−18.101
M06-2X/6-31++G(d,p)	−20.090
PW91/aug-cc-pVTZ	−18.370
PW91/6-31++G(d,p)	−20.209
Correlated methods	
	**System**
CCSD/aug-cc-pVDZ	−16.324
CCSD/aug-cc-pVTZ	−15.962
CCSD/6-31++G(d,p)	−18.417
DLPNO-CCSD(T_0)/aug-cc-pVDZ(loose PNO)	−24.228
DLPNO-CCSD(T_0)/aug-cc-pVDZ(normal PNO)	−24.350
DLPNO-CCSD(T_0)/aug-cc-pVDZ(tight PNO)	−24.321
DLPNO-CCSD(T_0)/aug-cc-pVTZ(loose PNO)	−25.131
DLPNO-CCSD(T_0)/aug-cc-pVTZ(normal PNO)	−25.287
DLPNO-CCSD(T_0)/aug-cc-pVTZ(tight PNO)	−25.412
DLPNO-CCSD(T_0)/aug-cc-pVQZ(loose PNO)	−34.115
DLPNO-CCSD(T_0)/aug-cc-pVQZ(normal PNO)	−34.255
DLPNO-CCSD(T_0)/aug-cc-pVQZ(tight PNO)	−34.436
DLPNO-CCSD(T_0)-F12/cc-pVDZ-F12 (loose PNO)	−23.643
DLPNO-CCSD(T_0)-F12/cc-pVDZ-F12 (normal PNO)	−23.923
DLPNO-CCSD(T_0)-F12/cc-pVDZ-F12 (tight PNO)	−24.076
DLPNO-CCSD(T_0)-F12/cc-pVTZ-F12 (loose PNO)	−23.984
DLPNO-CCSD(T_0)-F12/cc-pVTZ-F12 (normal PNO)	−24.392
DLPNO-CCSD(T_0)-F12/cc-pVTZ-F12 (tight PNO)	−24.492

Table S7: Binding free energies obtained at semi-empirical, DFT, and CCSD level of theory and interaction single-point energies obtained at CCSD(T_0) level of theory in kcal/mol for one water molecule with one chloride ion.

Semi-empirical methods	
	**System**
GFN1-xTB	171.447
GFN2-xTB	146.698
B97-3c	125.562
PBEh-3c	153.973
r^2SCAN-3c	149.040
DFT methodologies	
	**System**
ωB97X-D/aug-cc-pVTZ	129.849
ωB97X-D/6-31++G(d,p)	318.276
M06-2X/aug-cc-pVTZ	128.675
M06-2X/6-31++G(d,p)	130.292
PW91/aug-cc-pVTZ	113.294
PW91/6-31++G(d,p)	115.322
Correlated methods	
	**System**
CCSD/aug-cc-pVDZ	136.240
CCSD/aug-cc-pVTZ	132.507
CCSD/6-31++G(d,p)	132.460
DLPNO-CCSD(T_0)/aug-cc-pVDZ(loose PNO)	126.289
DLPNO-CCSD(T_0)/aug-cc-pVDZ(normal PNO)	126.295
DLPNO-CCSD(T_0)/aug-cc-pVDZ(tight PNO)	125.661
DLPNO-CCSD(T_0)/aug-cc-pVTZ(loose PNO)	122.832
DLPNO-CCSD(T_0)/aug-cc-pVTZ(normal PNO)	122.567
DLPNO-CCSD(T_0)/aug-cc-pVTZ(tight PNO)	121.812
DLPNO-CCSD(T_0)/aug-cc-pVQZ(loose PNO)	122.768
DLPNO-CCSD(T_0)/aug-cc-pVQZ(normal PNO)	122.378
DLPNO-CCSD(T_0)/aug-cc-pVQZ(tight PNO)	121.576
DLPNO-CCSD(T_0)-F12/cc-pVDZ-F12 (loose PNO)	127.344
DLPNO-CCSD(T_0)-F12/cc-pVDZ-F12 (normal PNO)	126.516
DLPNO-CCSD(T_0)-F12/cc-pVDZ-F12 (tight PNO)	126.556
DLPNO-CCSD(T_0)-F12/cc-pVTZ-F12 (loose PNO)	125.325
DLPNO-CCSD(T_0)-F12/cc-pVTZ-F12 (normal PNO)	125.008
DLPNO-CCSD(T_0)-F12/cc-pVTZ-F12 (tight PNO)	124.797

S3 Cluster Binding Free Energies and Hydrate Distributions

Table S8: Absolute Binding free energies in kcal/mol for the pyruvic acid-containing clusters.

DLPNO-CCSD(T_0)/aug-cc-pVTZ//ωB97X-D/6-31++G(d,p)			
Number of water molecules	$(PA)_1(Na^+)_1(w)_{0-5}$	$(PA)_1(Cl^-)_1(w)_{0-5}$	$(PA)_1(Na^+)_1(Cl^-)_1(w)_{0-5}$
0	−29.961	−20.469	−144.789
1	−37.057	−22.411	−152.774
2	−48.289	−21.276	−158.498
3	−51.621	−22.452	−159.188
4	−54.388	−23.303	−161.495
5	−57.011	−22.489	−162.390

Table S9: Absolute Binding free energies in kcal/mol for the pyruvate-containing clusters.

DLPNO-CCSD(T_0)/aug-cc-pVTZ//ωB97X-D/6-31++G(d,p)		
Number of water molecules	$(PA^-)_1(Na^+)_1(w)_{0-5}$	$(PA)_1(Na^+)_1(Cl^-)_1(w)_{0-5}$
0	−131.104	−173.330
1	−139.181	−173.975
2	−145.306	−179.229
3	−150.326	−183.330
4	−152.944	−185.957
5	−154.153	−187.815

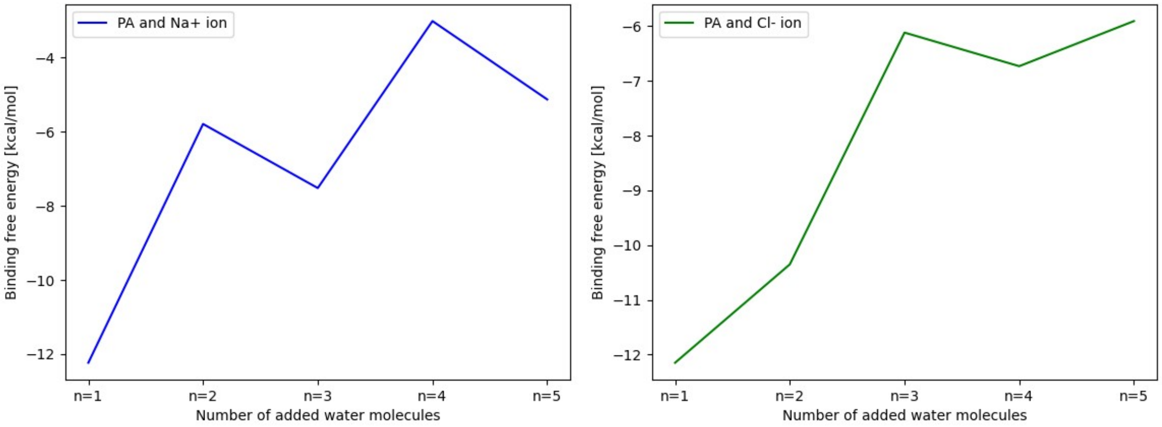

Figure S2: Binding free energy profiles of the competitive water displacement reactions of PA on the ionic clusters containing Na^+ (left panel) and Cl^- (right panel).

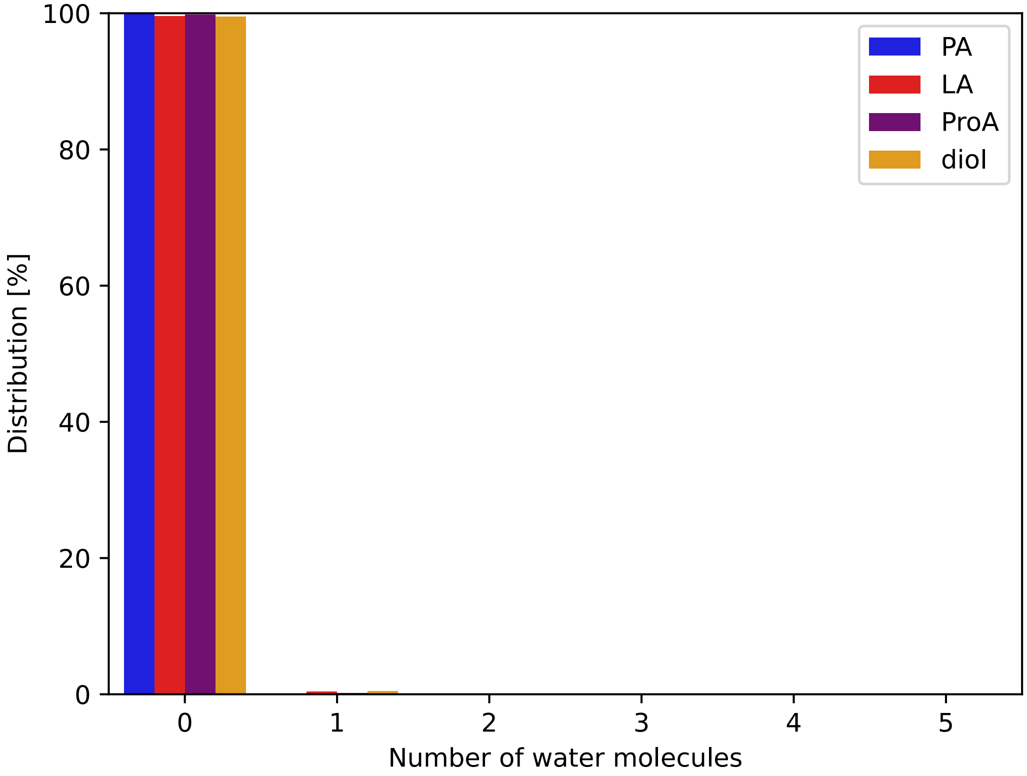

Figure S3: Equilibrium hydrate distribution of PA (blue), LA (red), ProA (purple), and diol (orange) at 273.15 K and 100% relative humidity.

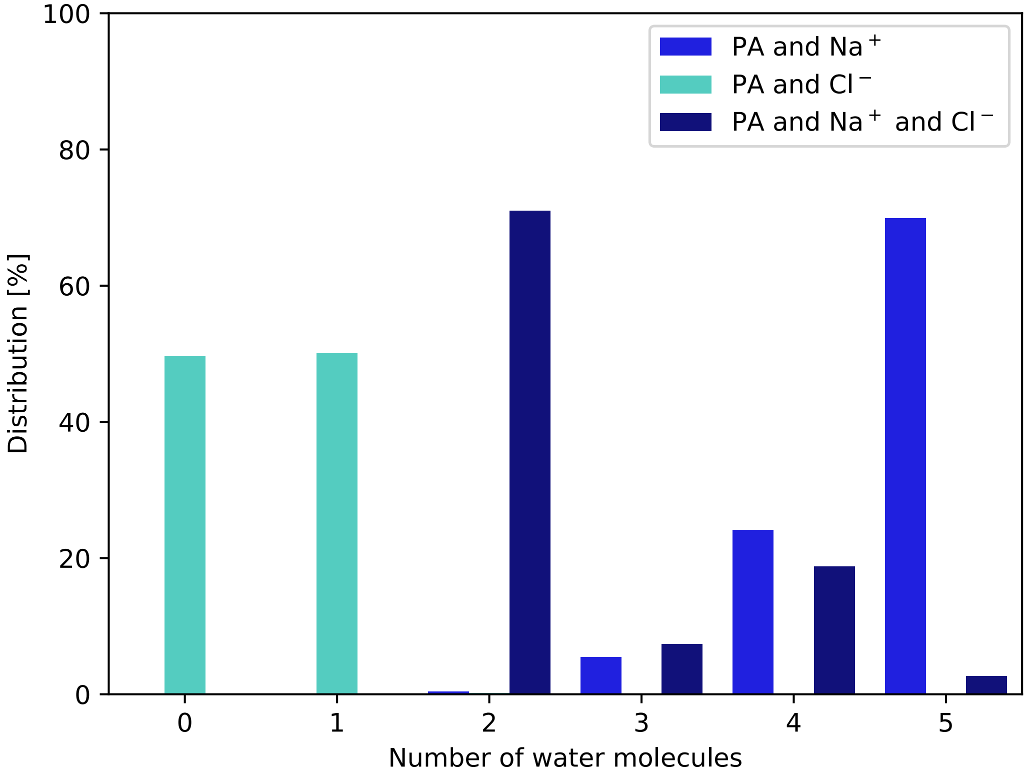

Figure S4: Equilibrium hydrate distribution of PA with Na^+ (blue), PA with Cl^- (turquoise), and PA with Na^+ and Cl^- (dark blue) at 273.15 K and 100% relative humidity.

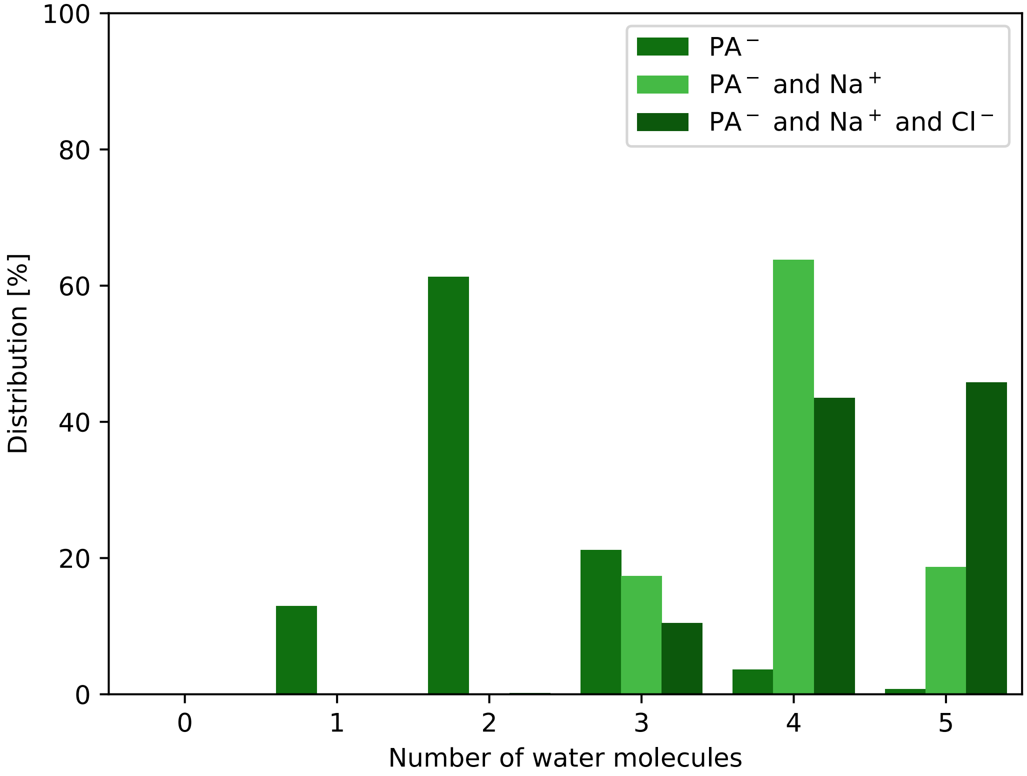

Figure S5: Equilibrium hydrate distribution of PA^- (green), PA^- with Na^+ (lime green), and PA^- with Na^+ and Cl^- (dark green) at 273.15 K and 100% relative humidity.

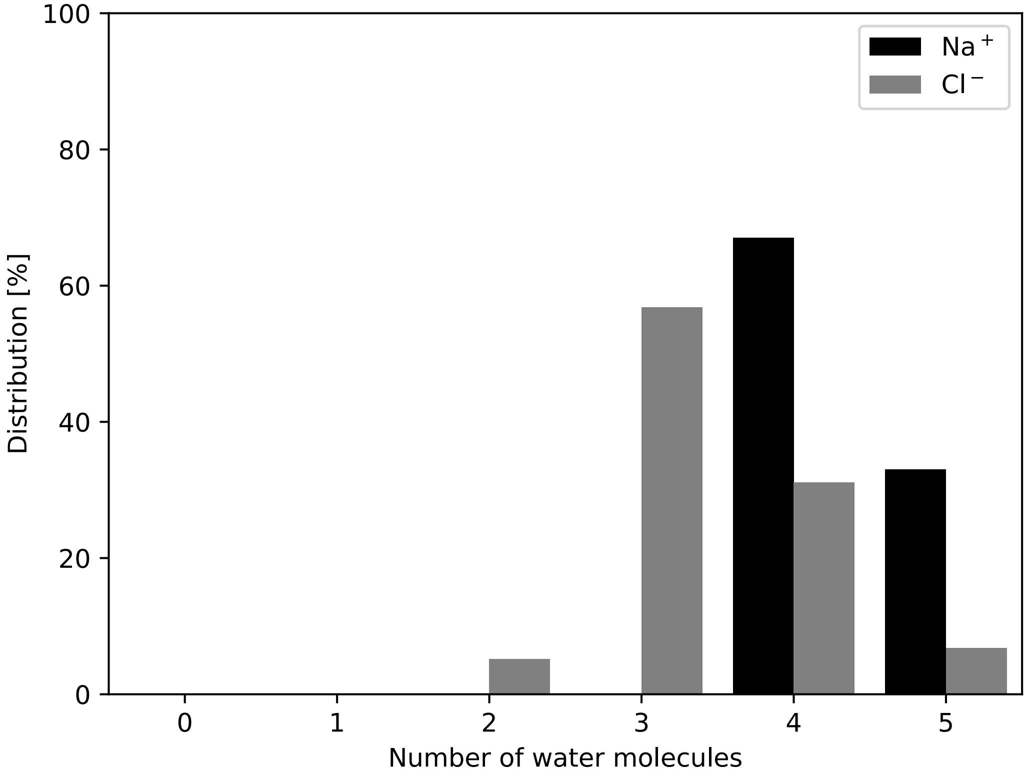

Figure S6: Equilibrium hydrate distribution of Na^+ (grey) and Cl^- (black) at 273.15 K and 100% relative humidity.

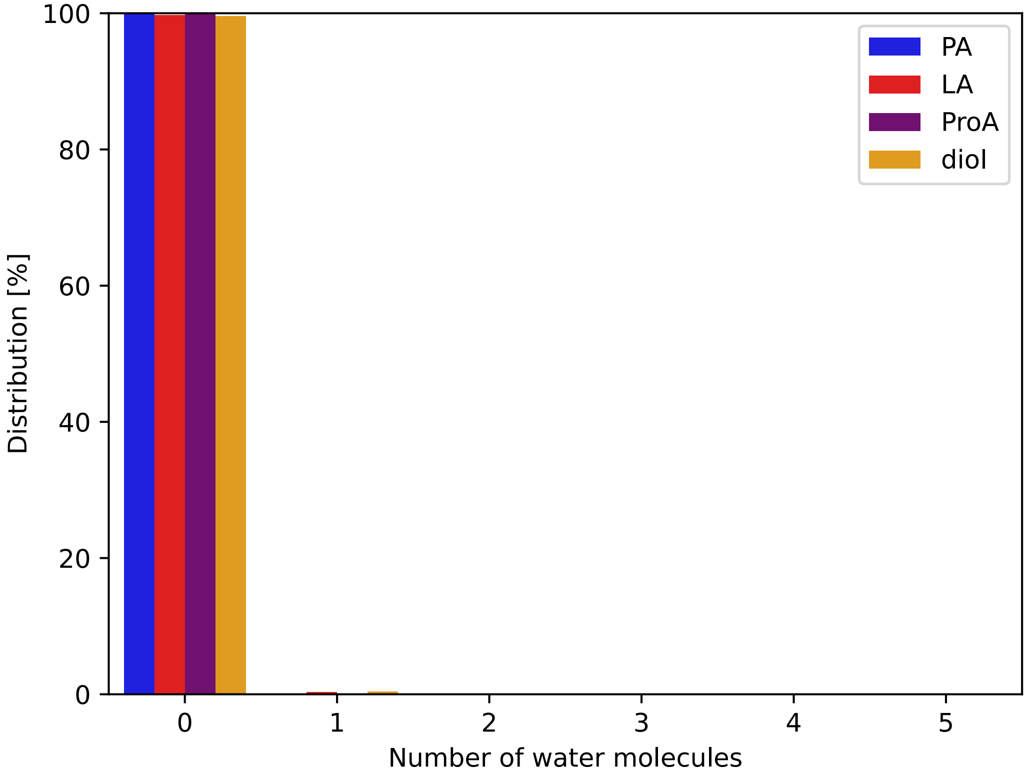

Figure S7: Equilibrium hydrate distribution of PA (blue), LA (red), ProA (purple), and diol (orange) at 258.15 K and 100% relative humidity.

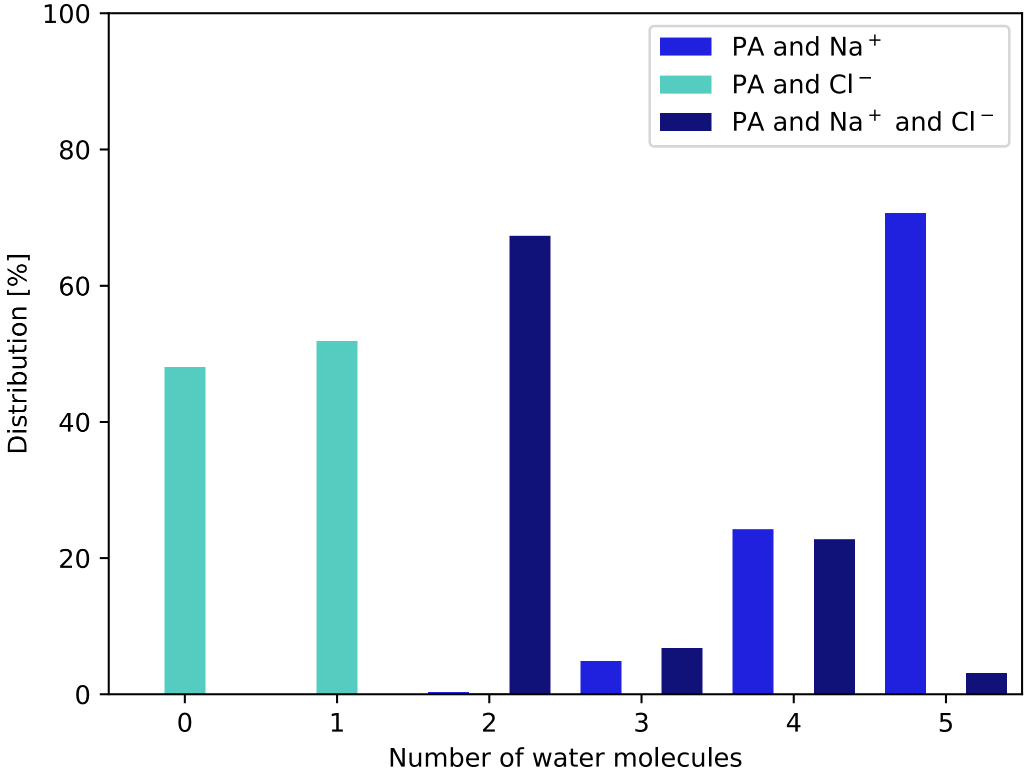

Figure S8: Equilibrium hydrate distribution of PA with Na^+ (blue), PA with Cl^- (turquoise), and PA with Na^+ and Cl^- (dark blue) at 258.15 K and 100% relative humidity.

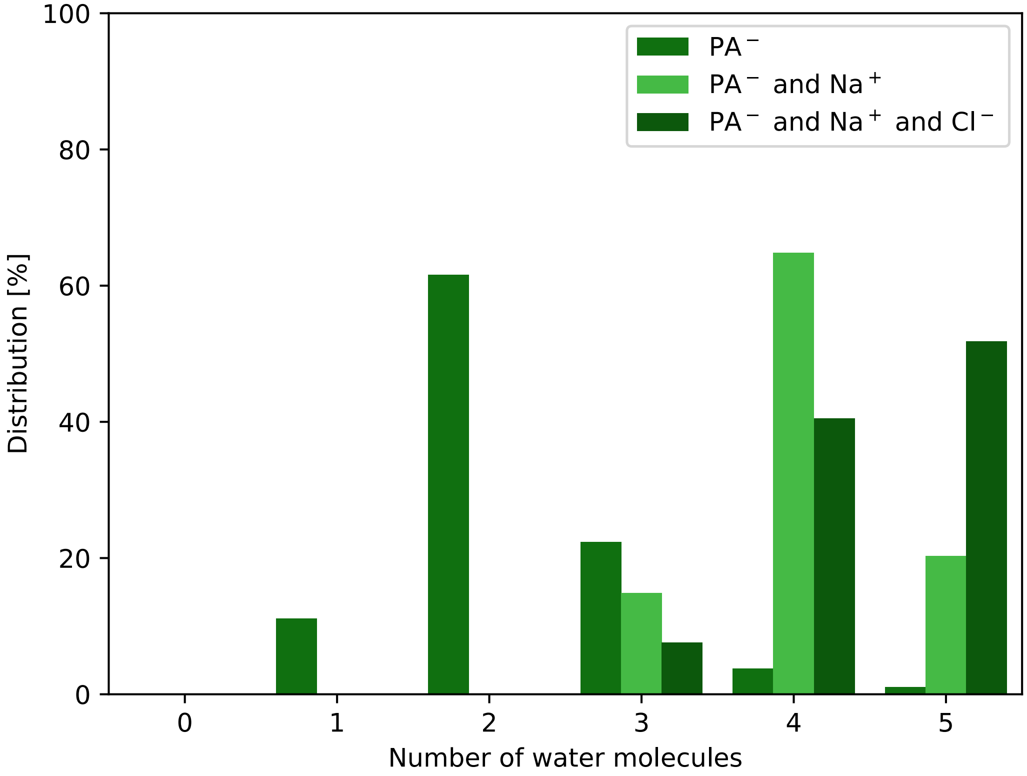

Figure S9: Equilibrium hydrate distribution of PA^- (green), PA^- with Na^+ (lime green), and PA^- with Na^+ and Cl^- (dark green) at 258.15 K and 100% relative humidity.

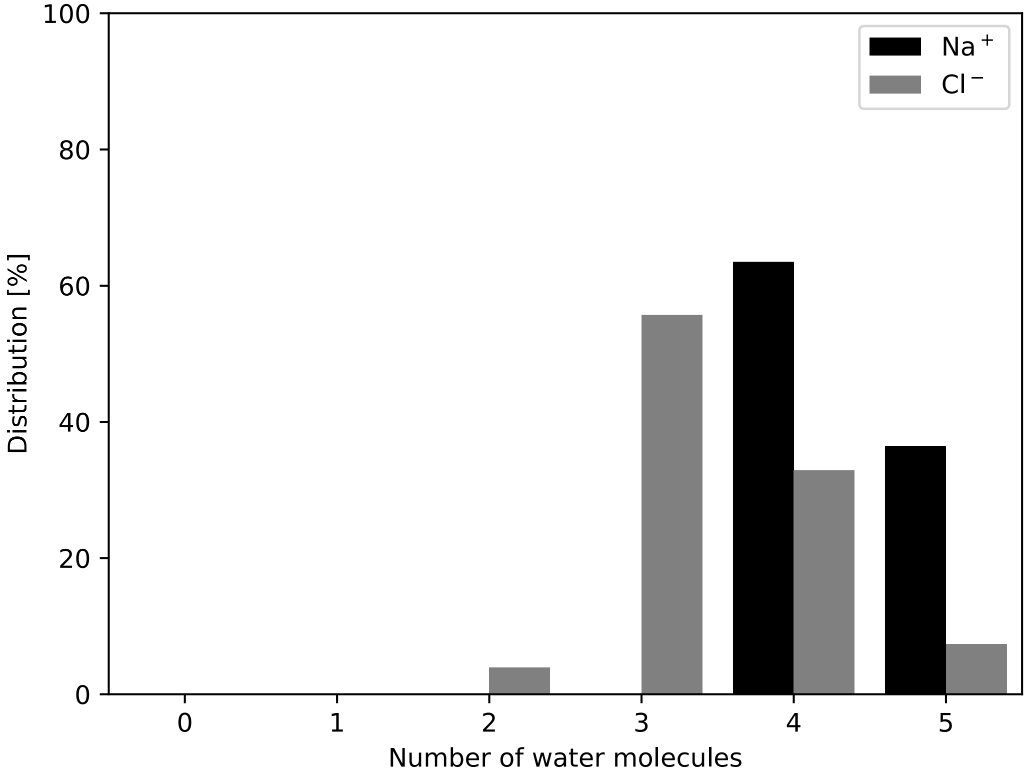

Figure S10: Equilibrium hydrate distribution of Na^+ (grey) and Cl^- (black) at 258.15 K and 100% relative humidity.

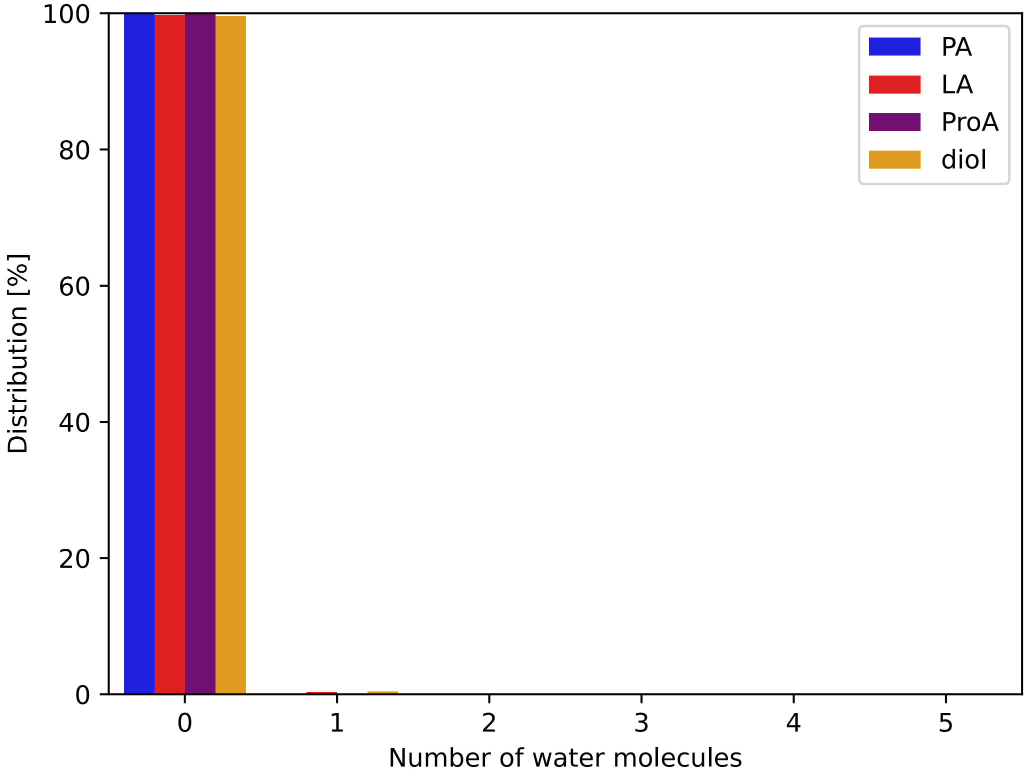

Figure S11: Equilibrium hydrate distribution of PA (blue), LA (red), ProA (purple), and diol (orange) at 298.15 K and 50% relative humidity.

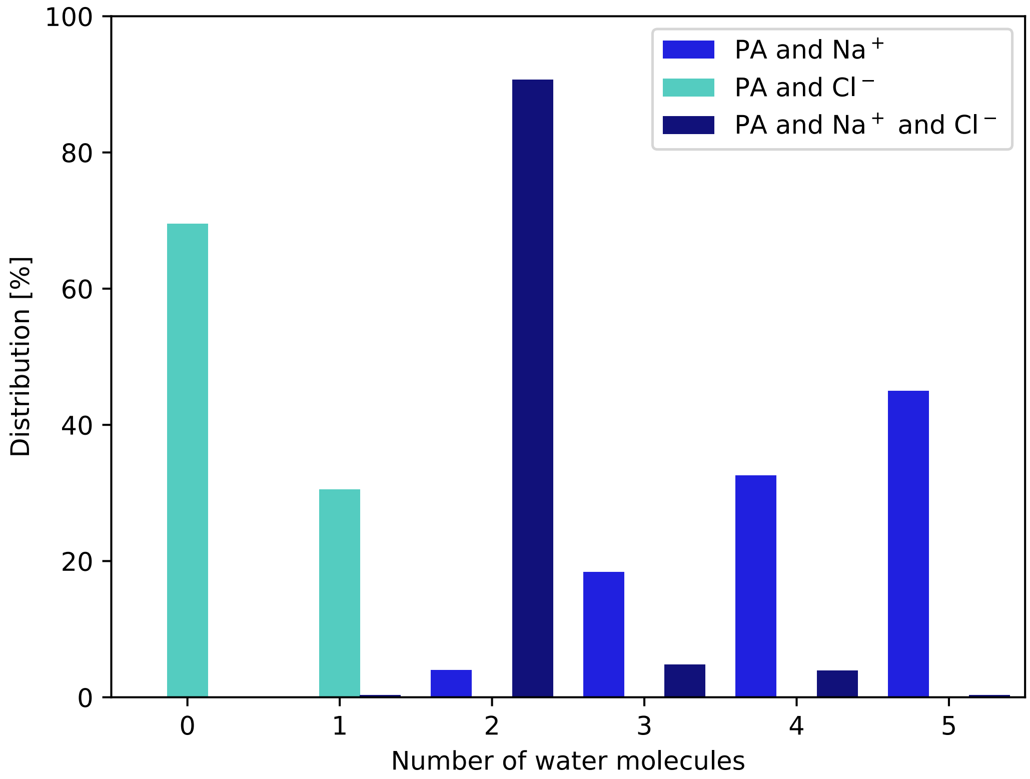

Figure S12: Equilibrium hydrate distribution of PA with Na^+ (blue), PA with Cl^- (turquoise), and PA with Na^+ and Cl^- (dark blue) at 298.15 K and 50% relative humidity.

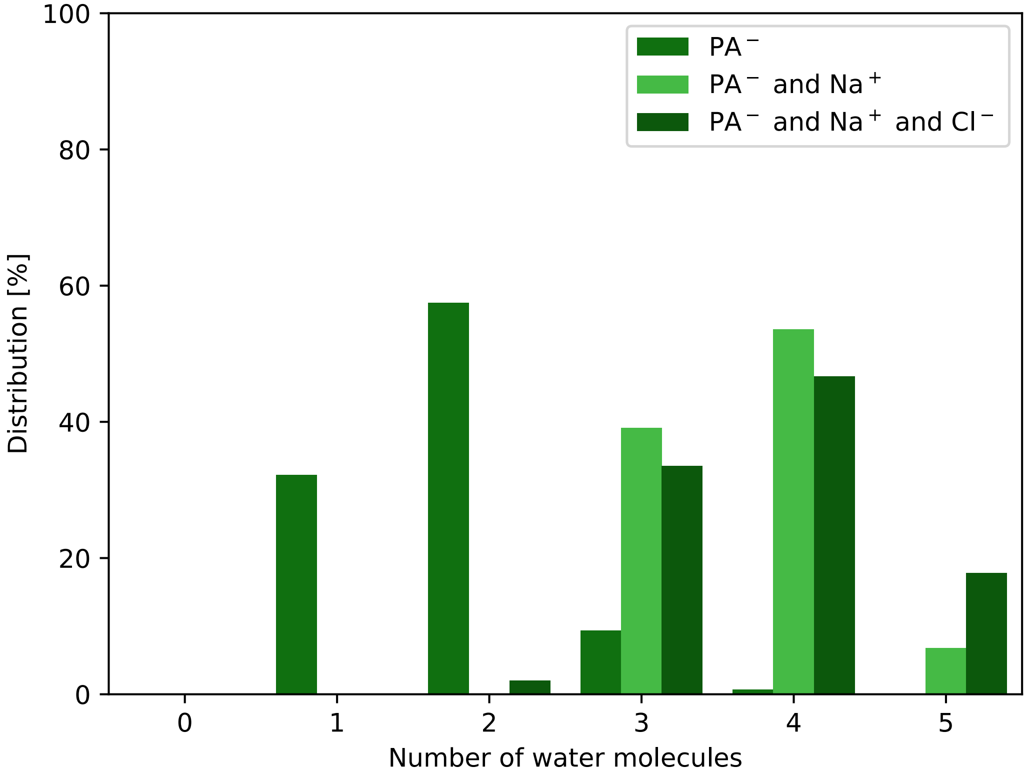

Figure S13: Equilibrium hydrate distribution of PA^- (green), PA^- with Na^+ (lime green), and PA^- with Na^+ and Cl^- (dark green) at 298.15 K and 50% relative humidity.

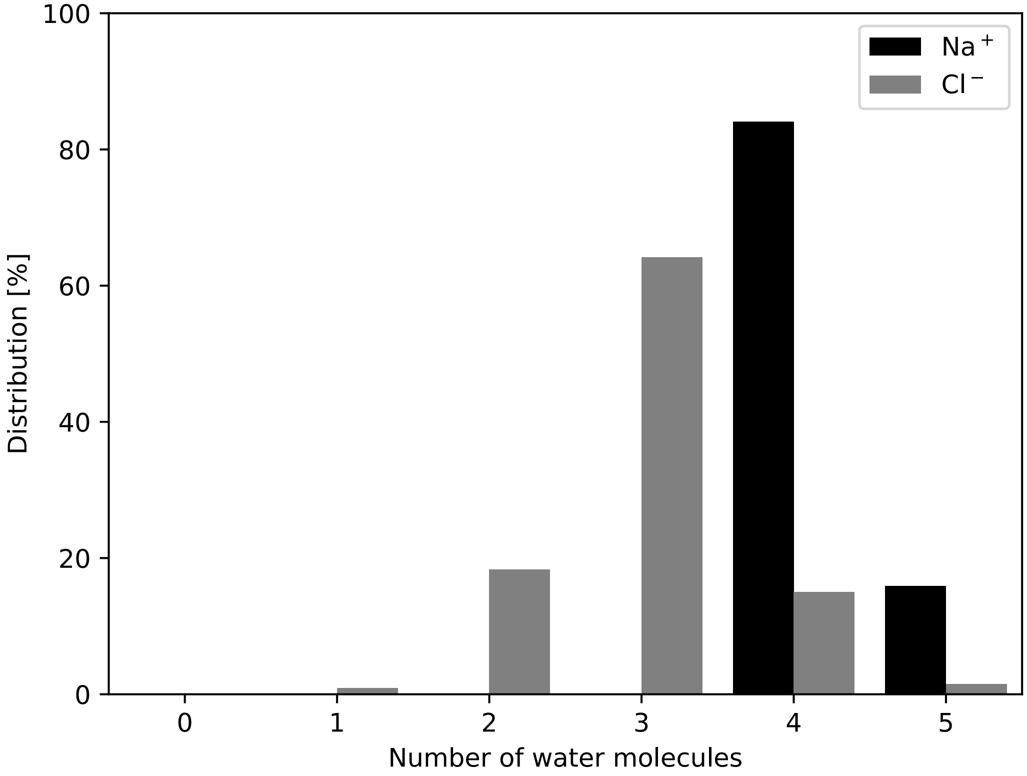

Figure S14: Equilibrium hydrate distribution of Na^+ (grey) and Cl^- (black) at 298.15 K and 50% relative humidity.

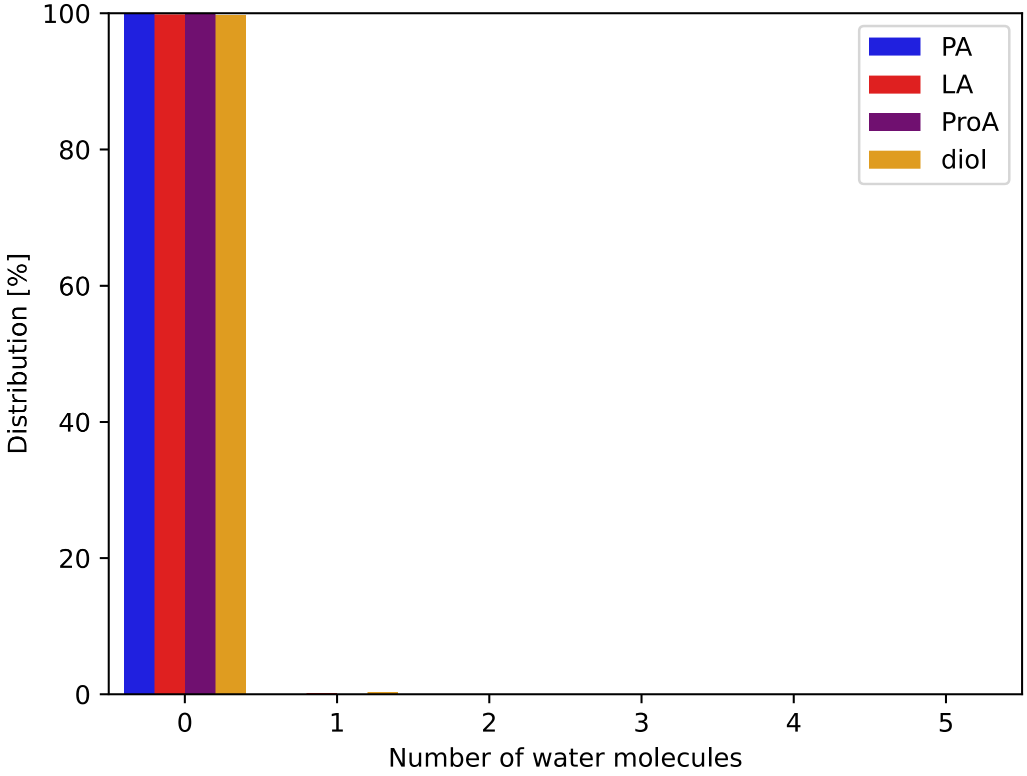

Figure S15: Equilibrium hydrate distribution of PA (blue), LA (red), ProA (purple), and diol (orange) at 273.15 K and 50% relative humidity.

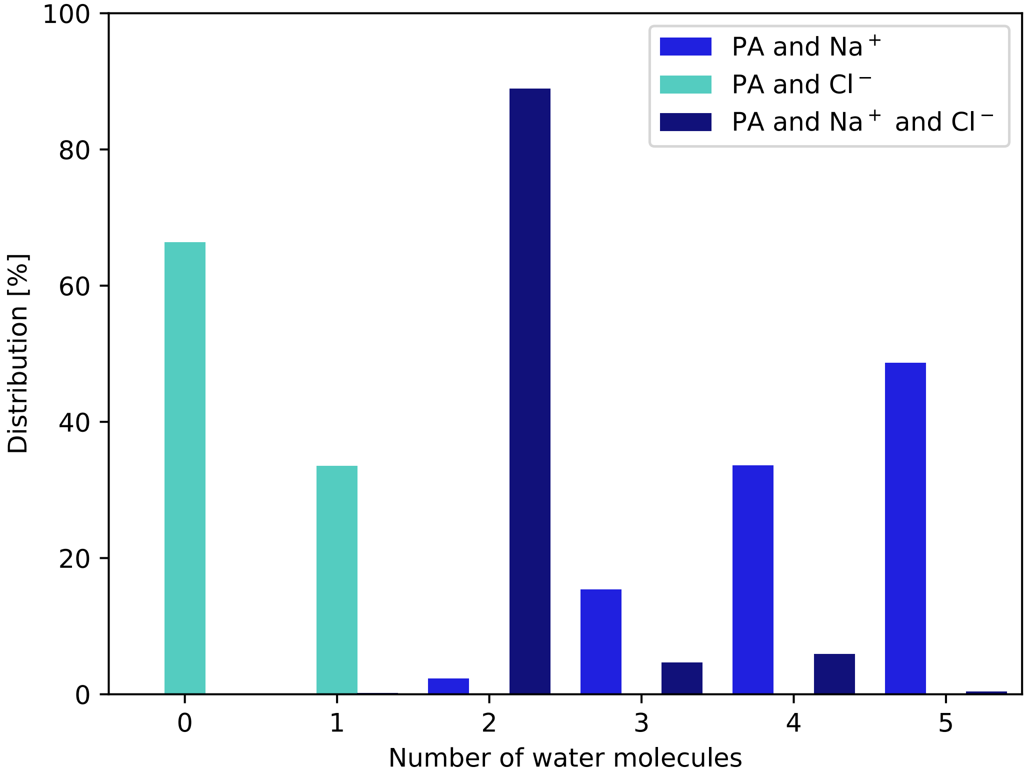

Figure S16: Equilibrium hydrate distribution of PA with Na^{+} (blue), PA with Cl^{-} (turquoise), and PA with Na^{+} and Cl^{-} (dark blue) at 273.15 K and 50% relative humidity.

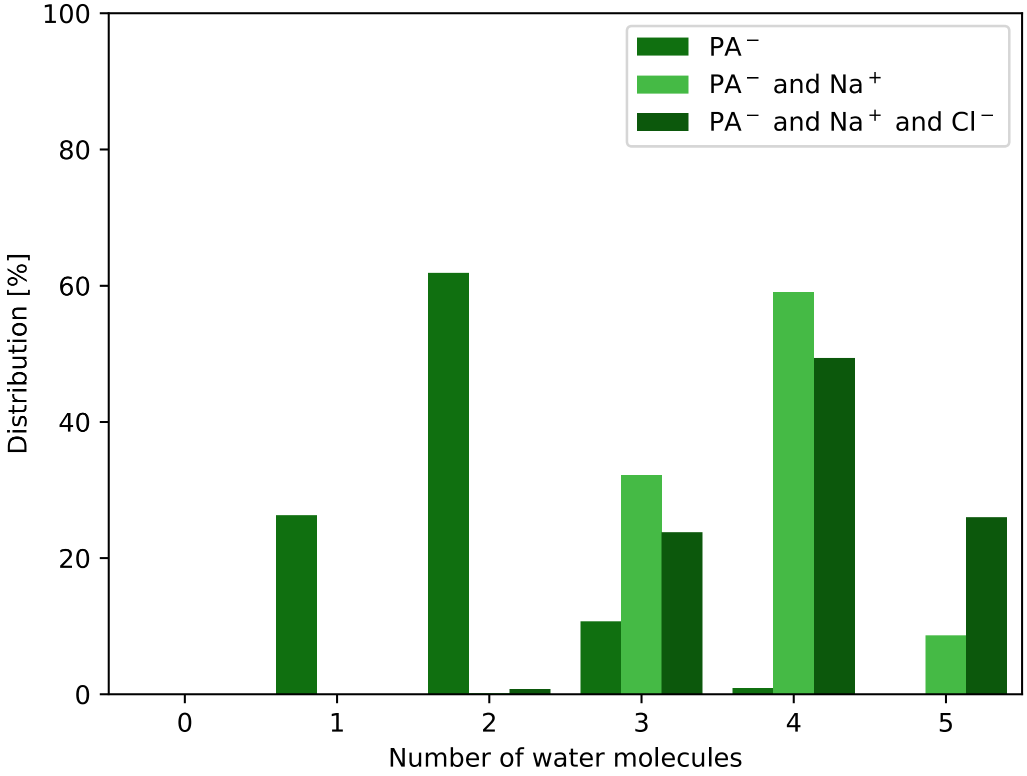

Figure S17: Equilibrium hydrate distribution of PA^- (green), PA^- with Na^+ (lime green), and PA^- with Na^+ and Cl^- (dark green) at 273.15 K and 50% relative humidity.

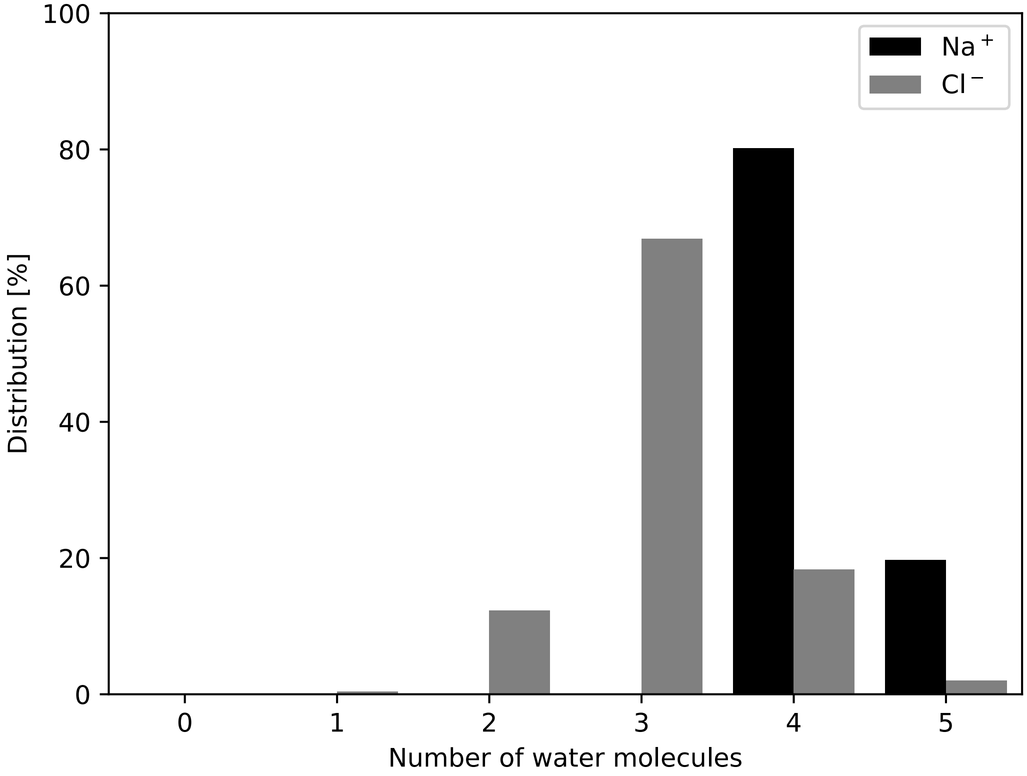

Figure S18: Equilibrium hydrate distribution of Na^+ (grey) and Cl^- (black) at 273.15 K and 50% relative humidity.

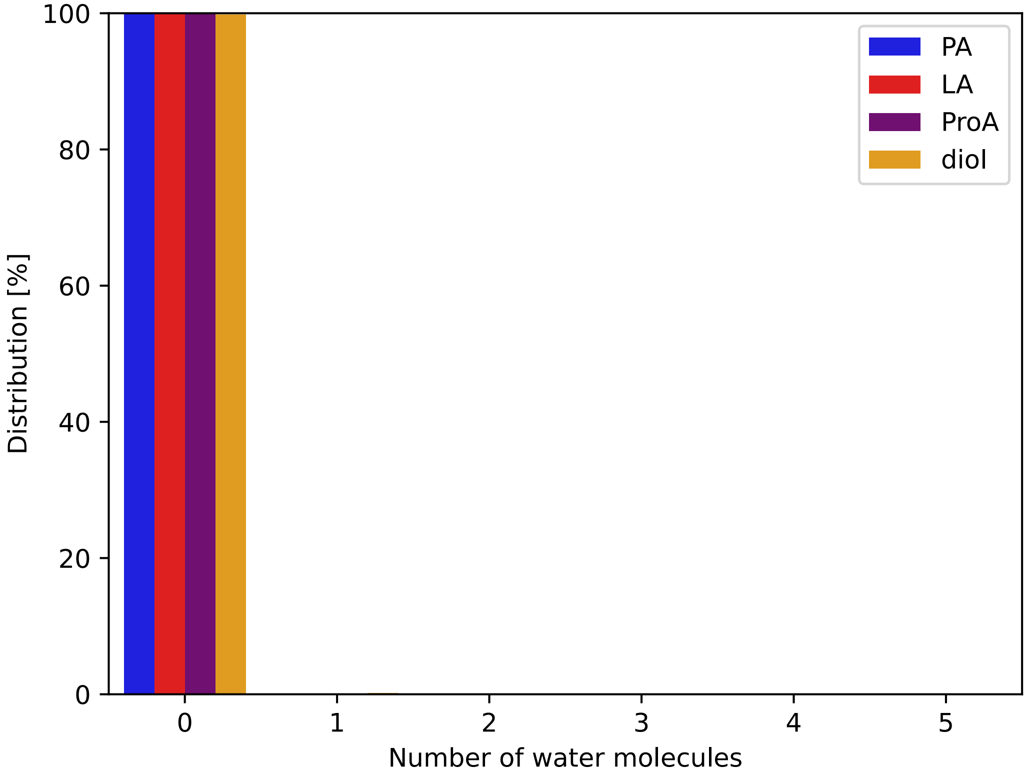

Figure S19: Equilibrium hydrate distribution of PA (blue), LA (red), ProA (purple), and diol (orange) at 258.15 K and 50% relative humidity.

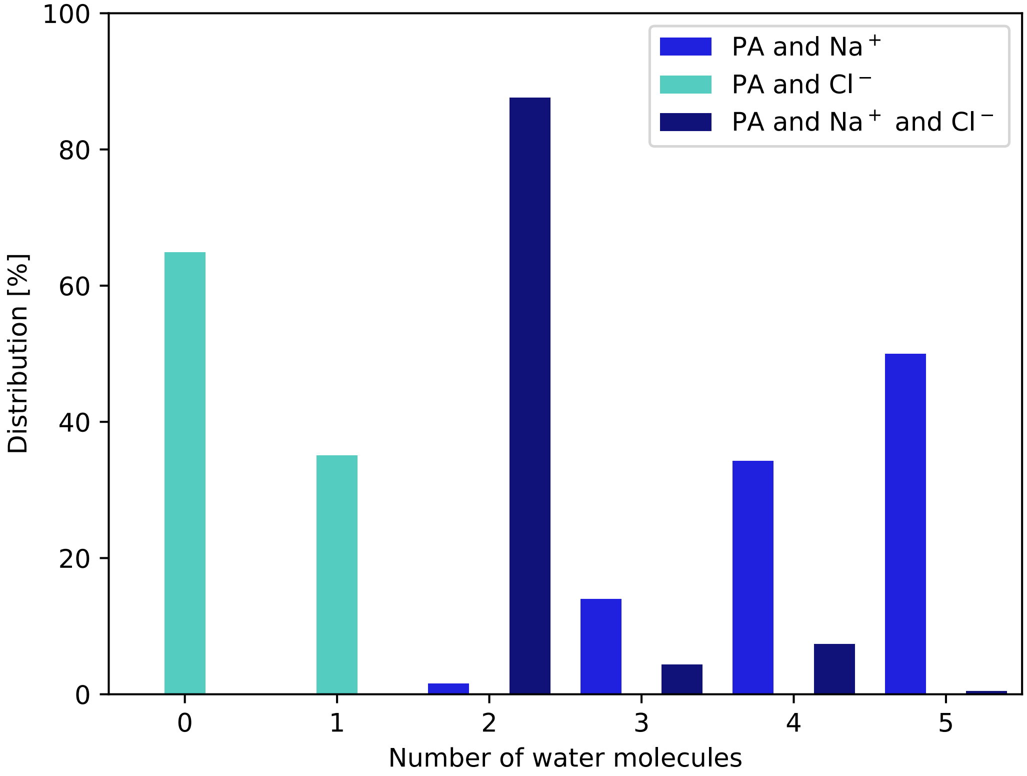

Figure S20: Equilibrium hydrate distribution of PA with Na^+ (blue), PA with Cl^- (turquoise), and PA with Na^+ and Cl^- (dark blue) at 258.15 K and 50% relative humidity.

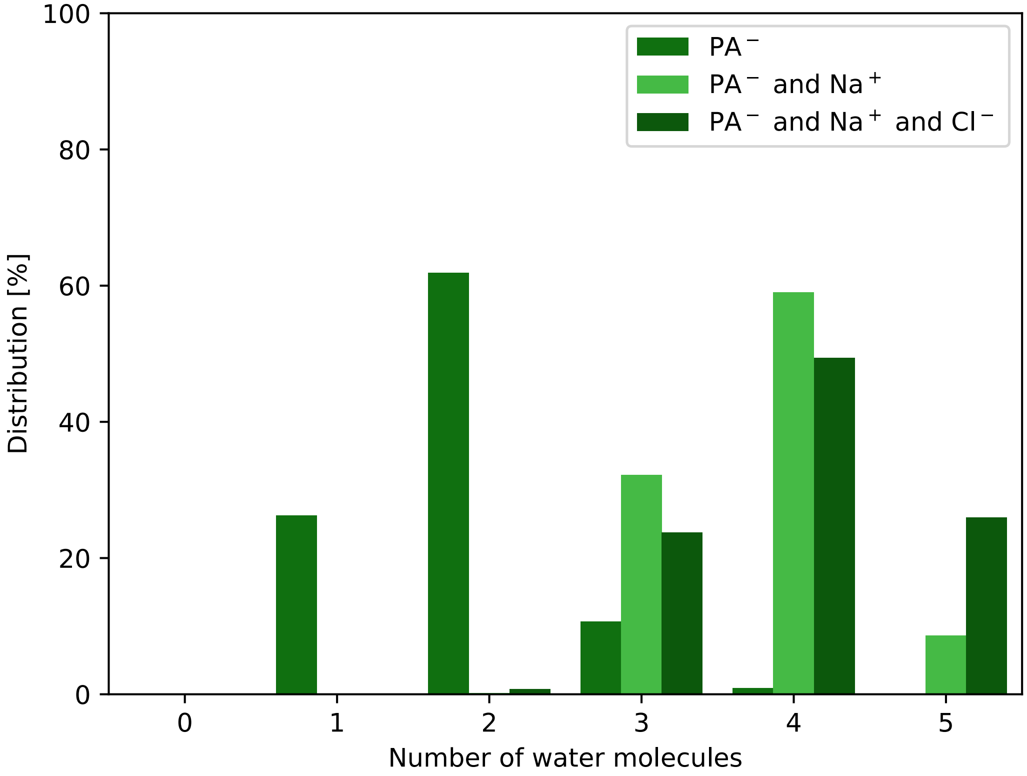

Figure S21: Equilibrium hydrate distribution of PA^- (green), PA^- with Na^+ (lime green), and PA^- with Na^+ and Cl^- (dark green) at 258.15 K and 50% relative humidity.

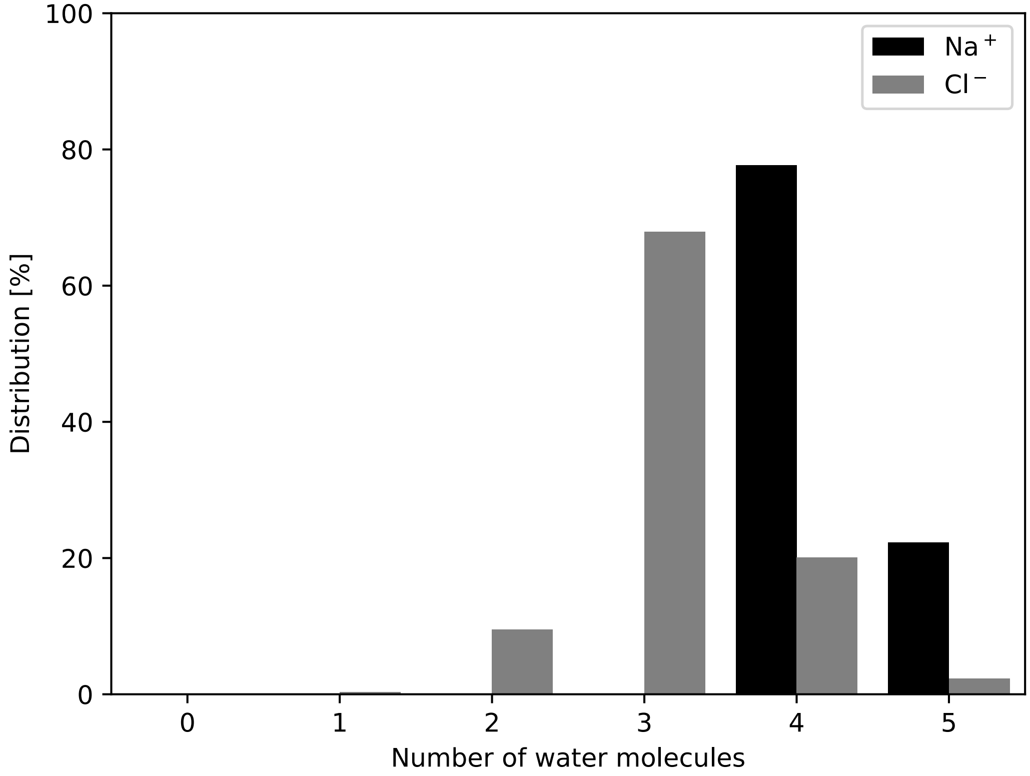

Figure S22: Equilibrium hydrate distribution of Na^+ (grey) and Cl^- (black) at 258.15 K and 50% relative humidity.

S4 Supporting Data and Structures

The coordinates of the calculated structures and the associated thermochemistry are available in the Atmospheric Cluster Database at: `https://github.com/elmjonas/ACDB/tree/master/Articles/trolle24_orgacids_w_na_cl`
